# Manipulating adrenergic stress receptor signalling to enhance immunosuppression and prolong survival of vascularized composite tissue transplants

**DOI:** 10.1002/ctm2.996

**Published:** 2022-08-22

**Authors:** Minhyung Kim, Daniel T. Fisher, Paul N. Bogner, Umesh Sharma, Han Yu, Joseph J. Skitzki, Elizabeth A. Repasky

**Affiliations:** ^1^ Department of Surgical Oncology Roswell Park Comprehensive Cancer Center Buffalo New York USA; ^2^ Department of Immunology Roswell Park Comprehensive Cancer Center Buffalo New York USA; ^3^ Department of Pathology Roswell Park Comprehensive Cancer Center Buffalo New York USA; ^4^ Department of Medicine, Division of Cardiology University at Buffalo Buffalo New York USA; ^5^ Department of Biostatistics and Bioinformatics Roswell Park Comprehensive Cancer Center Buffalo New York USA

**Keywords:** immunosuppression, stress signalling, vascularized composite tissue allotransplantation, β_2_‐adrenergic receptors

## Abstract

**Background:**

Vascularized composite tissue allotransplantation (VCA) to replace limbs or faces damaged beyond repair is now possible. The resulting clear benefit to quality of life is a compelling reason to attempt this complex procedure. Unfortunately, the high doses of immunosuppressive drugs required to protect this type of allograft result in significant morbidity and mortality giving rise to ethical concerns about performing this surgery in patients with non‐life‐threatening conditions. Here we tested whether we could suppress anti‐graft immune activity by using a safe β_2_‐adrenergic receptor (AR) agonist, terbutaline, to mimic the natural immune suppression generated by nervous system‐induced signalling through AR.

**Methods:**

A heterotopic hind limb transplantation model was used with C57BL/6 (H‐2b) as recipients and BALB/c (H‐2d) mice as donors. To test the modulation of the immune response, graft survival was investigated after daily intraperitoneal injection of β_2_‐AR agonist with and without tacrolimus. Analyses of immune compositions and quantification of pro‐inflammatory cytokines were performed to gauge functional immunomodulation. The contributions to allograft survival of β_2_‐AR signalling in donor and recipient tissue were investigated with β_2_‐AR^−/−^ strains.

**Results:**

Treatment with the β_2_‐AR agonist delayed VCA rejection, even with a subtherapeutic dose of tacrolimus. β_2_‐AR agonist decreased T‐cell infiltration into the transplanted grafts and decreased memory T‐cell populations in recipient's circulation. In addition, decreased levels of inflammatory cytokines (IFN‐γ, IL‐6, TNF‐α, CXCL‐1/10 and CCL3/4/5/7) were detected following β_2_‐AR agonist treatment, and there was a decreased expression of ICAM‐1 and vascular cell adhesion molecule‐1 in donor stromal cells.

**Conclusions:**

β_2_‐AR agonist can be used safely to mimic the natural suppression of immune responses, which occurs during adrenergic stress‐signalling and thereby can be used in combination regimens to reduce the dose needed of toxic immunosuppressive drugs such as tacrolimus. This strategy can be further evaluated for feasibility in the clinic.

## Graphical abstract

Enhancing immunosuppression by adrenergic receptor manipulation using a β_2_‐agonist enhances VCA graft survival without increasing toxicity.

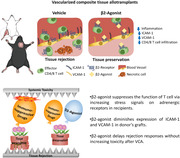

## 1 INTRODUCTION

Remarkable improvements in on‐site emergency care have led to an increased survival of patients with traumatic injuries that previously would have been fatal‐injuries which result from accidents, gun‐shots or explosions in both civilian and military settings.^1^, ^2^, ^3^ However, these surviving patients must endure life‐long difficulties associated with the loss of legs, arms, hands or other functional units (including faces) with a significant decline in quality of life. This difficult situation has generated interest in testing new transplant protocols using body parts from cadaveric donors in a procedure known as vascularized composite tissue allotransplantation (VCA). With more than 100 patients receiving VCA over the past decade, this novel transplantation is raising hope for patients with devastating deformities and complex tissue defects.^4^, ^5^, ^6^, ^7^, ^8^ In large part, these successes have been achieved both by improved microsurgical techniques (e.g. anastomoses of vessels and nerves) and the use of large doses of potent pharmacological agents to induce immunosuppression, including cyclosporine A, tacrolimus and mycophenolic acid.^9^, ^10^, ^11^, ^12^ However, despite the excitement surrounding VCA, significant challenges prevent its widespread acceptance and use. First and foremost is that tissues and organs recovered from cadaveric donors are a scare resource with little chance of a human leukocyte antigen (HLA)‐match with the recipient.^13^ As a result, very heavy doses of immunosuppressive medications are required, exposing the patient to opportunistic infections, hyperglycaemia, hepatotoxicity, nephrotoxicity, cancer and reproductive toxicity.^8^, ^14^, ^15^ Additionally, in vivo and in vitro studies suggest that the immunosuppressants cyclosporine and tacrolimus can promote carcinogenesis and cancer progression through production of transforming growth factor‐β, increasing tumour angiogenesis and metastasis.^16^, ^17^ Adverse reactions and toxicity often necessitate reduction, and even complete withdrawal, of immunosuppressive drugs leading to a tragic graft rejection and loss. As an example of this scenario, a patient who received the first face transplantation in 2005 suffered from two different types of cancer as a consequence of potent immunosuppression and subsequently lost her lips due to graft rejection.^18^ Although VCA can provide significant improvement in quality of life, far too many of these patients experience either graft rejection or increased risk of additional health problems, including cancer, from the chronic use of high dose immunosuppressive drugs.^14^ This has led to significant ethical concerns about using this type of transplant in patients when it is not medically ‘life‐saving’, unlike the situation for patients requiring solid organ transplants such as liver or heart, which are medically required to save their lives. Moreover, with solid organ transplants, there is usually an opportunity to plan ahead and achieve a donor HLA match. Overall, there is a serious and unmet medical need for new strategies to improve graft survival after VCA and lessen the risk of life‐threatening morbidities from toxic immunosuppressive drugs.

The nervous and immune systems have been found to interact closely in host defence and stress responses.^19^, ^20^, ^21^, ^22^, ^23^, ^24^ Although the relationship of the hypothalamus–pituitary–adrenal axis and cortisol has been well studied,^25^, ^26^ the natural role of the autonomic nervous system in regulating immune responses is receiving increased attention; sympathetic and parasympathetic nerves are found innervating immune organs and near immune cells throughout the body. Extensive research now shows that neurotransmitter interactions between norepinephrine (NE) and β‐adrenergic receptors (ARs) regulate the immune system.^27^, ^28^, ^29^

Recently, we have shown that β_2_‐AR signalling has an important role in immune regulation of CD8^+^ T cells and myeloid‐derived suppressor cells (MDSC).^30^, ^31^, ^32^, ^33^, ^34^ The strong, naturally occurring immunosuppressive potential of β‐AR signalling is consistent with our observations that adrenergic stress or addition of β‐AR agonists can suppress graft versus host disease (GVHD) following allogeneic bone marrow transplantation (BMT).^34^, ^35^, ^36^

These data led us to investigate whether providing a pharmacological agonist of β‐AR, thus mimicking the natural neuro‐immune axis, could be exploited to suppress immune responses following VCA and permit a reduction in the dose of more toxic immunosuppressant drugs such as tacrolimus. Here, we investigated the impact of targeting β_2_‐AR, using the β_2_‐agonist terbutaline, on graft rejection rate and immune contexture using wild type (WT) and β_2_‐AR‐knock‐out (KO) mice. We found that increased β_2_‐AR signalling results in delayed rejection responses in VCA recipients without detectable toxicity and this occurred through mechanisms involving suppression of pro‐inflammatory cytokines and chemokine as well as inhibition of endothelial adhesion molecules need for infiltration of effector T cells. Importantly, we were able to extend graft survival using a subtherapeutic dose of tacrolimus combined with β_2_‐AR agonist. Together, these data reveal a feasible pathway, which, following further pre‐clinical optimization, can be tested in patients receiving VCA or other types of allotransplants.

## 2 MATERIALS AND METHODS

### 2.1 Mice

Female C57BL/6 (H‐2^b^), C57BL/6 (H‐2^b^, CD45.1) and BALB/c (H‐2^d^) mice aged 7–8 weeks were purchased from Charles River (Kingston, NY) and The Jackson Laboratory (Bar Harbor, ME) as recipients and donors, respectively. β_2_‐AR KO mice on BALB/c and C57BL/6 background are bred in‐house from an established colony. Mice were fed a standard laboratory diet and housed under standard light and accommodation conditions. All animal experiments were done with the approval of Roswell Park Comprehensive Cancer Center Animal Care and Use Committee IACUC.

### 2.2 VCA surgery

All procedures were carried out under sterile conditions by one investigator (M.K.) as described in our previous published work.^37^ Briefly, a donor's abdominal aorta and femoral vein were used for revascularization with a recipient's common carotid artery and external jugular vein, respectively, using a non‐suture cuff technique. We used BALB/c background strain as donors and C57BL/6 background strain as recipients because we have revealed that a BALB/c strain had a higher anatomical mutation rate on the Circle of Willis than C57BL/6 strain.^37^

### 2.3 Drug treatments

Immunosuppression was induced in mice using tacrolimus (Sigma‐Aldrich, St. Louis, MO) in doses of 2 or 4 mg/kg (in DMSO; Sigma‐Aldrich, St. Louis, MO) injected subcutaneously with a micro syringe (Hamilton, Reno, NV) daily. The 15 µg/µl of tacrolimus concentration was prepared, and up to 6 µl of diluent was injected without notable toxicity. β_2_‐AR activation was achieved using daily intraperitoneal injections of 2‐mg terbutaline or .05‐mg bambuterol (200 µl in DPBS; Corning Inc., Corning, NY). The same DPBS was used for vehicles.

### 2.4 HR and BP measurement

A noninvasive blood pressure (BP) monitoring system (CODA, Kent Scientific Corporation, Torrington, CT) was used to measure heart rate (HR) and BP in mice.^38^ Mice were acclimated with the system for 10 days prior to initiating experimental measurements. The results were recorded 6 h after each β_2_‐agonist injection during the period of experiment.

### 2.5 Blood collection

Blood was collected from the right superficial temporal vein (STV) using a sterile 5‐mm animal lancet (Medipoint, Inc., Mineola, NY) after anaesthesia induction. The STV is a large vessel positioned posterior to the eye, which can be traced one eye length back and one eye width up from the sebaceous gland.^39^ Concentrations of tacrolimus were measured in plasma prepared from the blood samples, which were collected 24 h after previous tacrolimus injection by VITROS 5.1 FS (Ortho Clinical Diagnostics, Inc., Rochester, NY).

### 2.6 H&E and IHC staining

Following standard euthanasia, grafted tissue was harvested and fixed in 10% formaldehyde (Thermo Fisher Scientific, Waltham, MA), and then tissue was embedded in paraffin. Formalin fixed paraffin sections were cut at 4 µm, placed on charged slides and dried at 60°C for 1 h. Slides were cooled to room temperature and added to the Leica Bond RX, where they were deparaffinized with Bond Dewax Solution (Leica, Allendale, NJ) and rinsed in water. Bond Epitope Retrieval Solution 2 (Leica, Allendale, NJ) was used for target retrieval for 30 min. Slides were blocked using peroxide block from a Bond Polymer Refine Detection kit (Leica, Allendale, NJ) for 5 min. Slides were incubated with CD4 Antibody (Abcam, Cambridge, United Kingdom) at 1/1000 or CD8 (Abcam, Cambridge, United Kingdom) at 1/1000 or FOXP3 (Boster Biological Technology, Pleasanton, CA) at 1/50 for 20 min followed by Rabbit Envision (Agilent Technologies, Santa Clara, CA) for 30 min. Diaminobenzidine from the Bond Polymer Refine Detection kit (Leica, Allendale, NJ) was applied for 10 min for visualization. Slides were counterstained with haematoxylin from the Bond Polymer Refine Detection kit (Leica, Allendale, NJ) for 8 min then placed into water. After removing slides from the Bond they were dehydrated, cleared and cover‐slipped.

### 2.7 Immunofluorescence histology

OCT (Sakura Finetek, Tokyo, Japan)‐embedded tissue cryosections (9‐µm thick) were fixed at −20°C in methanol/acetone (3:1), blocked using 1% bovine serum and stained with primary antibodies anti‐mouse ICAM‐1, ICAM‐2, vascular cell adhesion molecule‐1 (VCAM)‐1 antibodies (BD Biosciences, San Jose, CA) and anti‐mouse CD31 antibody (Abcam, Cambridge, United Kingdom). Images of at least five consecutive fields (unit area of each field, .34 mm^2^) were captured by observers blinded to sample identity. Identical exposure times and image settings were used within each experiment. Images were analysed with ImageJ software (NIH, Bethesda, MD) for the determination of the relative fluorescence staining intensity; regions of interest were defined based on CD31 fluorescence, and each pixel in identified regions was assigned a fluorescence intensity value (based on a scale from 0 to 255).

### 2.8 Flow cytometry

Spleens were mechanically disrupted and directly passed through a 70‐µm nylon cell strainer (Alkali Scientific, Pompano Beach, FL) followed by lysing red blood cells with hypotonic lysis buffer (Gibco, Gaithersburg, MD). Single‐cell suspensions were created from whole tissue transplanted grafts using the Medimachine tissue disruption system (Becton, Dickinson, Franklin Lakes, NJ), followed by leukocyte isolation using Lymphoprep (Stemcell Technologies, Vancouver, Canada). Prepared cells were stained with different antibodies for extracellular and intracellular markers. Antibodies of CD45 (BUV395, clone; 30‐F11), CD45.1 (BUV395, clone; 20), CD45.2 (BV605, clone; 104), CD3 (Alexa Fluor 700, 17A2), CD4 (PerCp, clone; RM4.5), CD8 (Alexa Fluor 488, clone; 53‐6.7), CD25 (APC, clone; PC61), CD44 (V450, clone; IM7), CD62L (PE, clone; MEL‐14), Foxp3 (PE, clone; MF23), IFN‐γ (PE‐CF594, clone; XMG1.2), IL4 (APC, clone; 11B11) and IL17 (BV421, clone; TC11‐18H10) were used (BD Bioscience, San Jose, CA). Golgi stop, fixation and Permeabilization Kit (BD Bioscience, San Jose, CA) were used for staining intracellular cytokines. All data were collected on an LSRFortessa flow cytometer (BD Biosciences, San Jose, CA) and analysed with WinList 9.0 software (Verity Software House, Topsham, ME). The markers CD44 and CD62‐L were used to classify CD4^+^ and CD8^+^ T cells as naive (CD44^−^ CD62‐L^+^), central memory (CM) (CD44^+^ CD62‐L^+^) or effector (CD44^+^ CD62‐L^−^).^40^, ^41^ The gating strategies for flow cytometry were represented in Figure S1.

### 2.9 Luminex assay

Plasma was prepared from collected blood after a 20‐min centrifuge at 800 *g* without using a brake. Mouse 11‐plex cytokine and 9‐plex chemokine were performed by Flow and Imaging Cytometry Shared Resource, Luminex Division at Roswell Park Comprehensive Cancer Center per the manufacturer's instructions (Invitrogen, Carlsbad, CA).

### 2.10 Bone marrow chimeras

Chimeras were generated between BALB/c WT and β_2_‐AR KO mice as donors. Recipient mice were lethally irradiated with 8.0 Gy of total body irradiation (Cesium^137^ source). One day after irradiation, bone marrow (BM) was reconstituted with the intravenous injection via a tail vein of 10 × 10^6^ donor cells. Reconstituted mice were used 8 weeks after BMT.

### 2.11 Assessment of rejection grade

Gross rejection grades and pathology rejection grades were evaluated by M.K. and P.N.B., respectively, based on the Banff 2007 working classification of skin‐containing composite tissue allograft pathology.^42^

### 2.12 Statistical analysis

Comparisons between groups were performed using Student's *t* test, and statistical significance was accepted with *p* < .05. Also, Two‐way ANOVA was performed to compare the change of measurements over time between groups by testing the group by time interaction effects. Note that a only small number of following pairwise comparisons were conducted, which were pre‐planned and mutually complementary, so correction for multiple testing was not necessary. In addition, Pearson's correlation was used with 95% confidence interval to deliver *p* values in the correlation data. Log‐rank (Mantel–Cox) test was used for survival comparisons between groups using GraphPad Prism, Version 8 software (GraphPad Software, Inc., La Jolla, CA).

## 3 RESULTS

### 3.1 Safety and cross‐reactivity with tacrolimus of selective β_2_‐AR agonist

Our previous work has established a reliable and consistent platform using a pre‐clinical murine model of hind limb VCA^37^ to investigate novel therapies to prolong transplant survival. The transplanted graft consisted of skin, fat, muscle, bone and blood vessels (Figure S2A), a complex combination of tissues similar to those which are often used in VCA.

Although we know from the literature and its clinical safety profile that terbutaline is considered to be a safe drug, we wanted to be sure that there were no cardiac physiology problems generated by this β_2_‐AR agonist alone, or in combination with the drug tacrolimus, a drug that causes significant immunosuppression and commonly used in the transplant setting.^14^, ^43^ We assessed BP and HR with a non‐invasive tail cuff system.^44^ Pre‐transplanted recipient mice were acclimated to the device for 10 days prior to collecting readings. No statistical difference was detected in systolic/diastolic BP or HR between mice treated daily with β_2_‐AR agonist or vehicle over a course of 11 days (Figure S2B). We also measured plasma levels of tacrolimus in the mice treated with two different doses; full dose (fTac, i.e. a dose known to generally maintain allografts long term^37^) and half dose (hTac, i.e. a dose that only delays graft rejection) were measured. As expected, significantly higher drug concentrations were measured in the fTac injected group compared to the hTac group, but there was no significant difference in tacrolimus concentrations between mice treated with tacrolimus alone and mice treated with tacrolimus and β_2_‐AR agonist combination (Figure S2C). In addition, no changes of body weight and physical appearance were detected suggesting that it was safe to use terbutaline alone and in combination with tacrolimus without drug interaction.

### 3.2 Decreased rejection responses and T‐cell infiltration into grafts with β2‐AR agonist alone and in combination with tacrolimus

Next, we assessed the impact of β_2_‐AR agonist on VCA survival by evaluating the impact of terbutaline in recipients employing histological haematoxylin and eosin (H&E) and immunohistochemistry (IHC) stains performed on transplanted grafts 5, 7 and 10 days after VCA to measure rejection grades and CD4^+^ T, CD8^+^ T and Foxp3^+^ cell infiltration into grafts.

**FIGURE 1:**
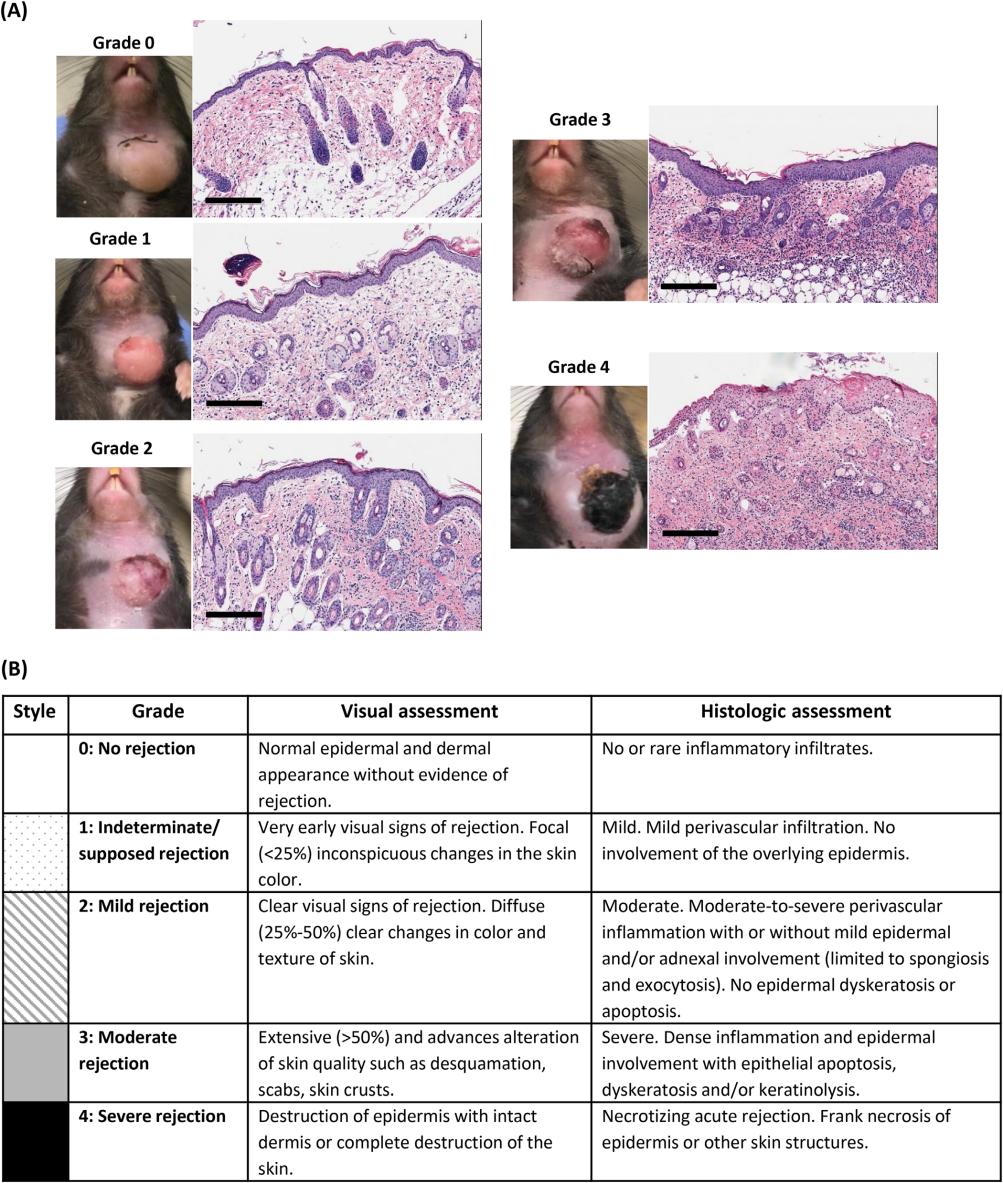
Visual and histologic grading systems for assessment of rejection after vascularized composite tissue allotransplantation (VCA). (A) Examples of each clinical and histologic rejection grade in a murine heterotopic hind limb transplant performed in a total major histocompatibility complex (MHC)‐mismatch. Scale bar: 50 µm. (B) The Banff 2007 working classification of skin‐containing composite tissue allograft pathology

Gross and histologic VCA rejections were graded using the Banff 2007 working classification of skin‐containing composite tissue allograft pathology (Figure 1A,B).^42^

**FIGURE 2:**
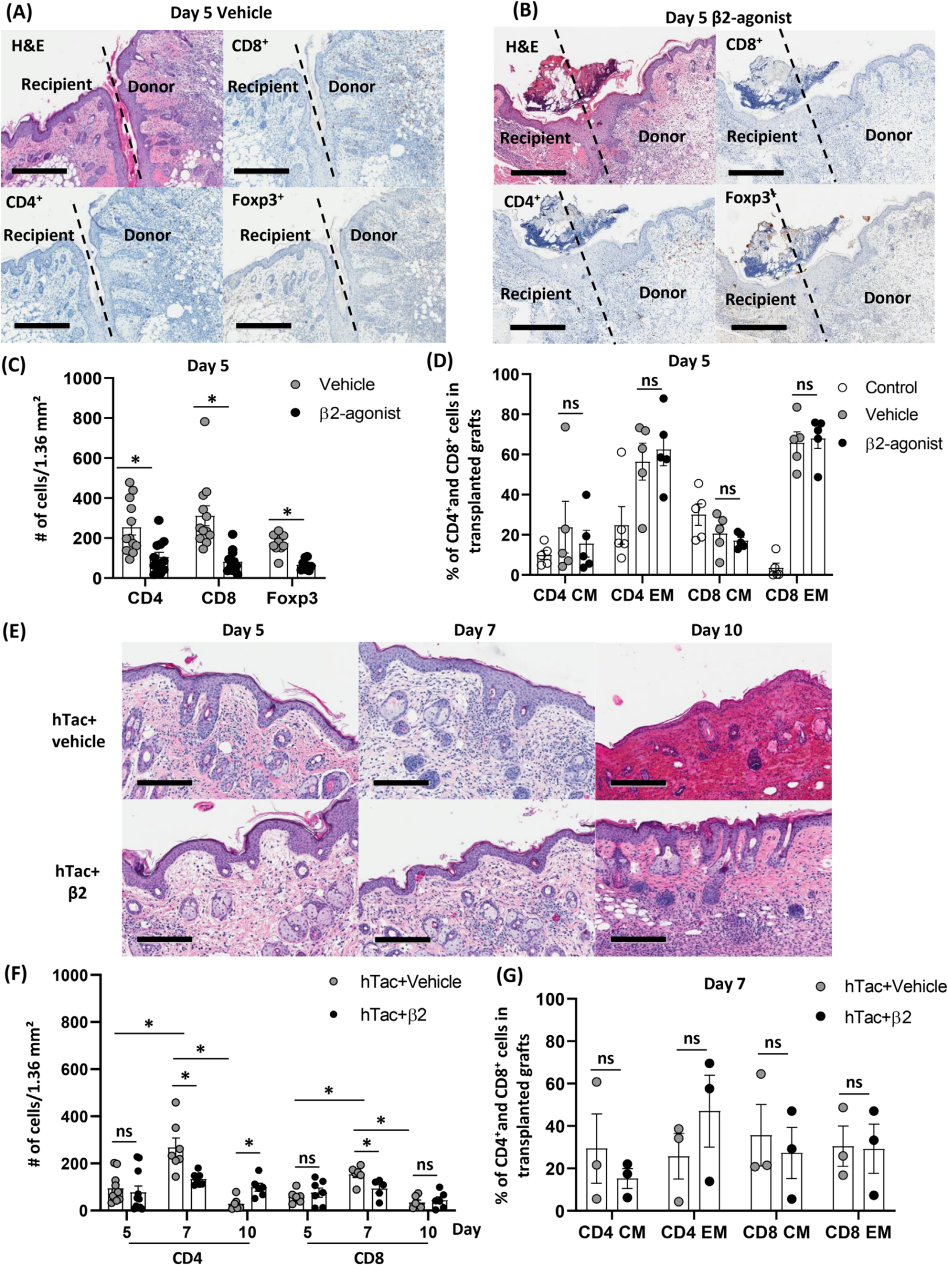
β_2_‐Adrenergic receptors (ARs) agonist decreases T‐cell infiltration in transplanted grafts along with lower numbers of Foxp3 positive cells compared to the vehicle‐injected group. (A and B) Representative figures for haematoxylin and eosin (H&E) and immunohistochemistry (IHC) with CD8, CD4, and Foxp3 antibodies with either vehicle or β_2_‐agonist treatment for 5 days. The borderline (black dotted line) differentiates recipient and donor, scale bar: 400 µm. (C) Numbers of CD4, CD8 and Foxp3 positive cells in grafts 5 days after vascularized composite tissue allotransplantation (VCA). Over eight fields from three mice/group. Error bar, standard error of the mean. *p < .05 by Student's *t* test. (D) Compositions of CD4^+^/CD8^+^ central memory (CM) and effector memory (EM) T‐cell populations in transplanted grafts 5 days after VCA. Control, non‐vascularized donor grafts; *n* = 5. ns, not significant; error bar, standard error of the mean. *p < .05 by Student's *t* test. (E) Representative figures for H&E stain at different time points after VCA. Scale bar: 200 µm. (F) Numbers of CD4^+^ and CD8^+^ T cells in grafts 5, 7 and 10 days after VCA in the hTac + vehicle and the hTac + β_2_‐agonist‐treated groups. Over five fields from *n* = 3/group. Error bar, standard error of the mean. *p < .05 by Student's *t* test. (G) Compositions of CD4^+^/8^+^ CM and EM T‐cell populations in transplanted grafts 7 days after treatment either with hTac or hTac + β_2_‐agonist. *n* = 3. ns, not significant; error bar, standard error of the mean

Reduced lymphocyte infiltration into the graft at an early phase of rejection would be expected to correlate with the observed enhanced graft survival. Although moderate perivascular inflammation was detected at day 5 in the β_2_‐agonist‐treated group (pathology grade 2), epithelial apoptosis and dyskeratosis were distinct in the vehicle‐treated group (pathology grade 3) (Figures 2A,B and S3A,B). Infiltrating lymphocytes (CD4^+^ T, CD8^+^ T and Foxp3^+^ cells) were concentrated in the donor, but not recipient tissue, in both vehicle (Figures 2A, S4A–S6A) and β_2_‐agonist (Figures 2B, S4B–S6B)‐treated mice; however, fewer lymphocytes infiltrated the grafts in the β_2_‐agonist group (Figure 2C). Although significantly less T‐cell infiltration was detected in β_2_‐agonist treated grafts, compositions of CD4^+^/CD8^+^ CM and effector memory (EM) T‐cell populations did not show a statistical difference by flow cytometry 5 days after VCA in the grafts (Figure 2D). By day 7, the histological delineation between epidermis and dermis was lost and tissue rejection was nearly complete in the vehicle (Figure S3C) compared to the β_2_‐agonist group (Figure S3D) and infiltrated CD4^+^ T, CD8^+^ T and Foxp3^+^ cells found in vehicle controls were declining resulting in no statistical difference with the β_2_‐agonist‐treated group (Figure S3E). The majority of T cells in the grafts 7 days after VCA were EM cells in both groups, and these values were higher than healthy donor (pre‐transplanted grafts) control specimens (Figure S3F).

To test whether we could use a β‐AR agonist to mimic natural NE and β‐AR interactions and reduce the dose of tacrolimus needed for immunosuppression, we tested tacrolimus at half dose (hTac) in combination with either vehicle or β_2_‐agonist. Grade 4 graft rejection was observed 10 days after VCA in the hTac + vehicle group, whereas the hTac + β_2_ group showed mainly only grade 2 with partial grade 3 rejection with intact skin histology (Figure 2E). Abundant CD4^+^ T and CD8^+^ T‐cell infiltration was present 7 days after VCA in the hTac + vehicle group, and then the values dropped significantly 3 days later. β_2_‐Agonist treatments significantly decreased the number of infiltrating CD4^+^ T and CD8^+^ T cells at day 7 compared to the hTac + vehicle group, but more CD4^+^ T‐cell infiltration was found in the β_2_‐agonist‐treated group than the vehicle injected group at day 10 representing remnant immune responses in the graft (Figure 2F). There was no statistical difference in the proportion of CM and EM T cell populations in grafts 7 days after VCA between two groups using hTac (Figure 2G). Significant interaction effects by two‐way ANOVA also suggest that increase in the number of infiltrating CD4^+^ T and CD8^+^ T cells from day 5 to 7 is attenuated by the β_2_‐agonist treatments (Table S1). This finding demonstrated that β_2_‐agonist significantly decreased T‐cell infiltration in transplanted grafts compared to vehicle injection after VCA, which is the likely basis for prolonged survival of the grafts.

### 3.3 Changes in memory T and Th1 cells with a β_2_‐AR agonist treatment in recipients

It was important to clarify the origin of the infiltrated lymphocytes in the transplanted grafts for applying a target therapy. To determine whether infiltrating cells originated from the donor or the recipient, we took advantage of differential CD45 isoform usage of C57BL/6 recipient (CD45.1) and BALB/c donor (CD45.2) mice. By day 7 post‐VCA, over 90% of leukocytes within grafts (Figure S7A) and peripheral blood (Figure S7B) were from recipients.

**FIGURE 3:**
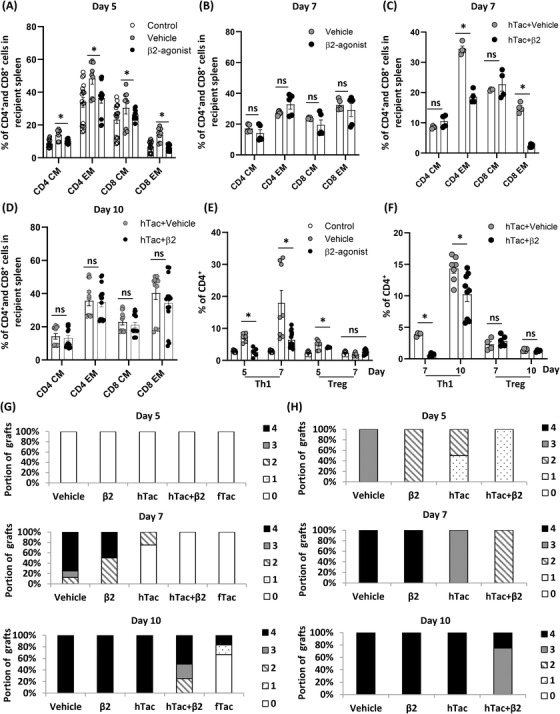
β_2_‐Adrenergic receptor (AR) agonist decreases CD4^+^/CD8^+^ effector memory (EM) T‐ and Th1‐cell populations and significantly decreases cytokine levels such as IFN‐γ, IL‐6 and TNF‐α compared to the vehicle group in recipients’ blood. (A and B) Compositions of CD4^+^/CD8^+^ central memory (CM)/EM T‐cell populations in the blood 5 and 7 days either with vehicle or β_2_‐agonist after vascularized composite tissue allotransplantation (VCA) without tacrolimus. Day 5, *n* ≥ 9; day 7, *n* = 5; control, non‐transplanted animals. ns, not significant; error bar, standard error of the mean. *p < .05 by Student's *t* test. (C and D) Compositions of CD4^+^/CD8^+^ CM/EM T‐cell populations in the blood 7 and 10 days with either vehicle or β_2_‐agonist after VCA with a half dose of tacrolimus (hTac). Day 7, *n* = 4; day 10, *n* ≥ 12. ns, not significant; error bar, standard error of the mean. *p < .05 by Student's *t* test. (E and F) Th1‐ and Treg‐cell populations in CD4^+^ T cells without and with tacrolimus after VCA with either vehicle or β_2_‐agonist treatments. Day 7, *n* ≥ 6; day 10, *n* ≥ 4. ns, not significant; error bar, standard error of the mean. *p < .05 by Student's *t* test. (G) Gross (clinical) rejection grades depending on different treatments after VCA. Vehicle, *n* = 8; β2, *n* = 4; hTac, *n* = 4; hTac + β2, *n* = 4; fTac, *n* = 6. (H) Histologic rejection grades depending on different treatments after VCA, *n* = 4/group

The frequency of different lymphocyte populations was analysed with either vehicle or β_2_‐agonist injections to investigate effects on recipients. In contrast to observations within grafts by flow cytometry (Figure 2D,G), β_2_‐AR agonist treatment significantly decreased the representation of CD4^+^ and CD8^+^ memory (CM and EM) T‐cell populations in the recipient's systemic blood compartment (spleen) at day 5 (i.e. before the emergence of signs of rejection; Figure 3A); however, by day 7 (i.e. once gross signs of rejection are apparent) these differences were lost (Figure 3B). This finding was more pronounced in the EM T‐cell population in mice treated with hTac. Additional decreases in the CD4^+^ T and CD8^+^ T EM populations were found with β_2_‐agonist treatment at day 7 (Figure 3C), and these differences are lost by day 10 (Figure 3D). β_2_‐Agonist decreased the compositions of Th1 population significantly compared to the vehicle group at day 5 and 7 without tacrolimus (Figure 3E) and at day 7 and 10 with hTac (Figure 3F). Interestingly, higher Treg (CD4^+^CD25^+^Foxp3^+^) levels in the graft (Figure 2C) and the body (spleen; Figure 3E) did not predict a better prognosis for transplanted grafts. There were more Foxp3^+^ cell infiltration in grafts and a greater Treg population systemically along with more infiltrated T cells in grafts. The Treg population was relatively proportional to the severity of T‐cell infiltration in grafts after VCA (Figure S8A,B). Accordingly, a smaller Treg population was found in grafts and the recipients treated with fTac (Figure S8C,D). Thus, Treg populations, which are thought to have immunosuppressive effects, may have increased as a consequence of a fulminant immune response to the highly antigenic VCA.

No gross rejection was observed in any graft prior to 5 days post‐VCA, but over 85% of the vehicle injected recipients showed grade 3 or 4 rejection 7 days after VCA. In contrast, β_2_‐AR‐agonist‐treated recipient mice had less severe rejection (grade 2) at day 7, which was further improved by the addition of subtherapeutic dose of tacrolimus (hTac), with some grafts surviving with grade 2 rejection at day 10 (Figure 3G). Although no evidence of gross rejection was observed at day 5, various histologic rejection grades were detected with H&E. β_2_‐Agonist treatments delayed rejection responses with/without subtherapeutic dose of tacrolimus compared to the vehicle‐injected group (Figure 3H). The data demonstrate that stimulation of β_2_‐AR decreases the presence of effector T‐ and Th1‐cell populations in recipients along with delayed visual and histologic evidence of rejection, and more Foxp3^+^ cells appear when more T cells exist in the graft representing severe rejection responses after VCA.

### 3.4 Analyses of inflammatory cytokine levels after β_2_‐AR modulation in recipients

**FIGURE 4:**
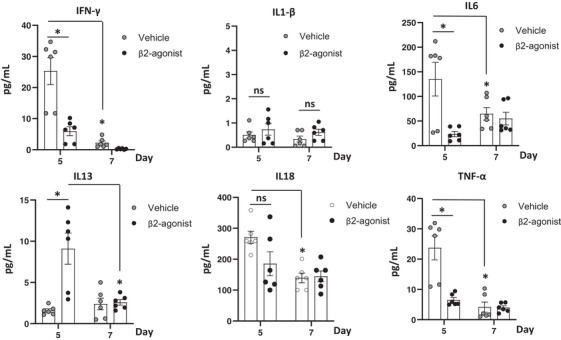
β_2_‐Adrenergic receptor (AR) modulation decreases inflammatory cytokine productions in recipients. In the setting of wild‐type (WT) donors and WT recipients vascularized composite tissue allotransplantation (VCA) without tacrolimus, various cytokine levels were analysed 5 and 7 days after transplant by Luminex assay. *n* = 6; pooled data from duplicate samples. Error bar; standard error of the mean. *p < .05 by Student's *t* test

The helper T cell (Th) is one of predominant populations releasing cytokines.^45^ β_2_‐Agonist decreased the frequency of Th1 significantly, but it was important whether manipulating of β_2_‐AR with agonists suppressed the production of cytokines. Luminex analyses were used to determine if differences in the expression of inflammatory cytokines are associated with graft rejection after VCA followed by vehicle or β_2_‐agonist injections. Systemic cytokines in recipient's plasma were measured. Significantly decreased IFN‐γ, IL‐6 and TNF‐α levels with increased IL‐13 levels were found in the β_2_‐agonist group compared to the vehicle group 5 days after VCA. The elevated cytokines (IFN‐γ, IL‐6, IL‐18 and TNF‐α) decreased 2 days later was indicative of their likely role in graft rejection responses before phenotypic rejection was noted (Figure 4), and the two‐way ANOVA showed that changes of cytokine levels from day 5 to 7 were statically different between two groups in IFN‐γ, IL‐6, IL‐13 and TNF‐α (Table S2). This finding suggests that β_2_‐agonist decreases the production of proinflammatory and inflammatory cytokines in recipients after VCA.

### 3.5 Augmentations of subtherapeutic immunosuppression using tacrolimus in combination with β_2_‐AR agonist

**FIGURE 5:**
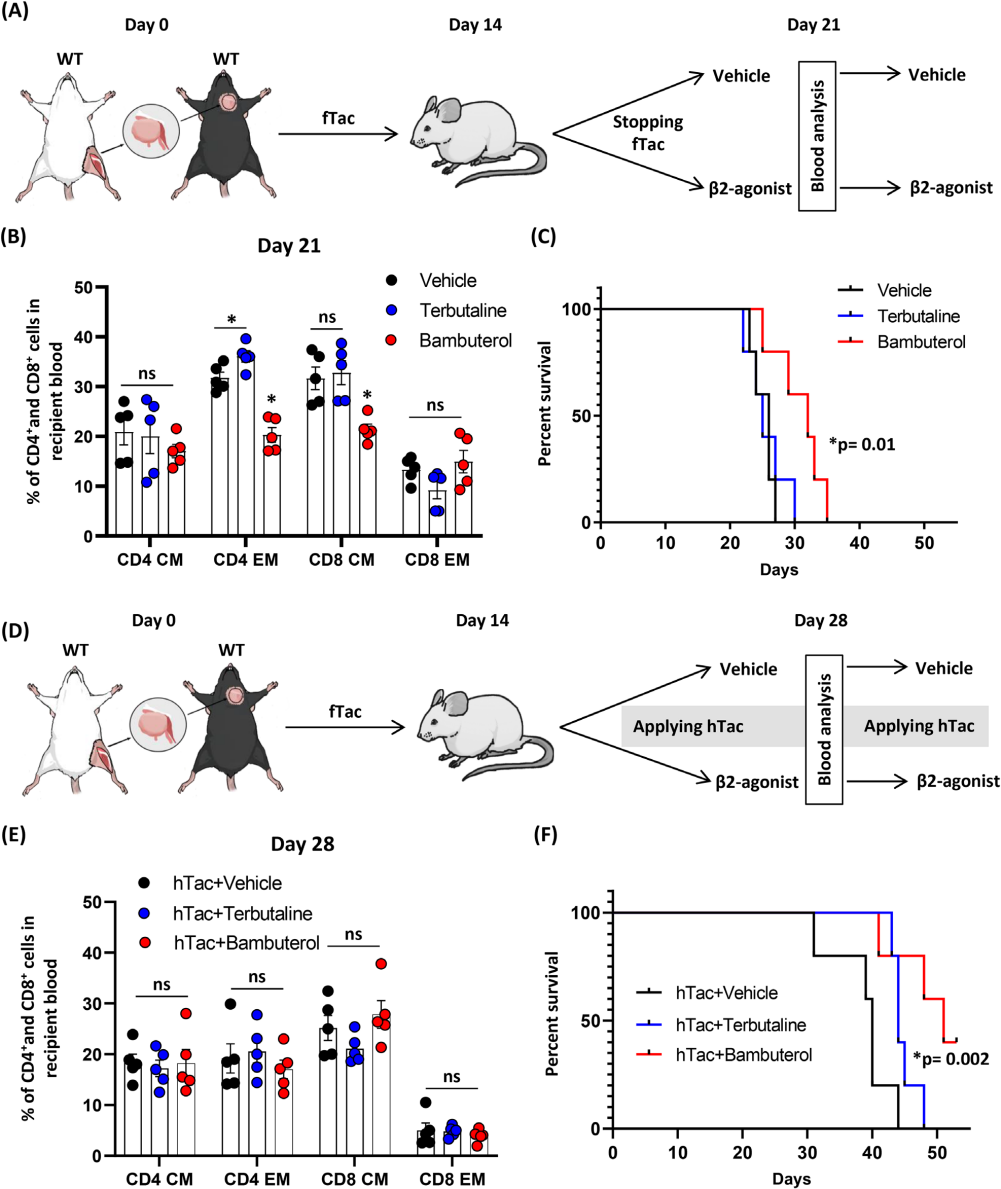
β_2_‐Adrenergic receptor (AR) agonist injected group achieves significantly longer graft survival than the vehicle injected group. (A) Wild type (WT) recipients having WT donor grafts were treated with a full dose (optimal) of tacrolimus (fTac) for 14 days and then treated with either vehicle or β_2_‐agonists (terbutaline and bambuterol) after cessation of fTac. (B) CD4^+^/CD8^+^ central memory (CM) and effector memory (EM) T‐cell compositions were analysed in the blood 21 days after vascularized composite tissue allotransplantation (VCA), 7 days after treatment with either vehicle or β_2_‐agonist. *n* = 5. ns, not significant; error bar, standard error of the mean. *p < .05 by Student's *t* test. (C) Graft survival curves. *n* = 5. ns, not significant. *p < .05 by log‐rank test using GraphPad Prism. (D) WT recipients were treated with a half dose (subtherapeutic) of tacrolimus along with vehicle or β_2_‐agonists (terbutaline and bambuterol) after 14‐day fTac injections. (E) CD4^+^/CD8^+^ CM and EM T‐cell compositions were analysed in the blood 28 days after VCA, 14 days after treatment with either vehicle or β_2_‐agonist. *n* = 5. ns, not significant by Student's *t* test; error bar, standard error of the mean. (F) Graft survival curves. *n* = 5. *p < .05 by log‐rank test with GraphPad Prism

VCA recipients often must accept a reduction or a cessation of immunosuppressive drugs after various lengths of time because of toxic side effects. We investigated whether addition of β_2_‐agonist could allow a reduced dose of a standard immunosuppressive drug such as tacrolimus. Two different β_2_‐AR agonists as a short‐ and long‐acting compounds with terbutaline and bambuterol, respectively, were evaluated for their effects on the survival of grafts after cessation of immunosuppression. Recipients were given with fTac every day for 14 days, and then tacrolimus injection was stopped, and vehicle or β_2_‐agonist injections were initiated and continued until grafts showed rejection (Figure 5A). Distinct effects of a short‐acting β_2_‐agonist treatment, terbutaline, on suppression of memory T‐cell populations were not detected, but a long‐acting β_2_‐agonist, bambuterol, suppressed the compositions of CD4 EM and CD8 CM populations significantly compared to the vehicle group in the blood 21 days after transplant (Figure 5B). Even though there was no survival benefit of grafts given a short‐acting β_2_‐agonist following cessation of tacrolimus (Figure 5C), bambuterol improved graft survival significantly compared to other two groups (Figure 5C). In addition, with a scenario using hTac with either vehicle or β_2_‐agonists (Figure 5D), the dose of tacrolimus was reduced by half 14 days after a full dose of tacrolimus daily treatment (fTac to hTac). Even though no statistical difference on CD4^+^ and CD8^+^ memory T‐cell populations was detected between three groups in the blood 28 days after transplant (Figure 5E), there was a significant graft survival benefit in the β_2_‐agonist groups using a short‐ and long‐acting compounds compared to the vehicle group (Figure 5F). These studies reveal that although a β_2_‐agonist cannot replace a conventional immunosuppressive drug, tacrolimus, as a single agent, their addition is able to extend graft survivals and delay rejection responses when recipients are not given the full dose of tacrolimus.

### 3.6 Effects of β_2_‐AR modulation in donor tissue

**FIGURE 6:**
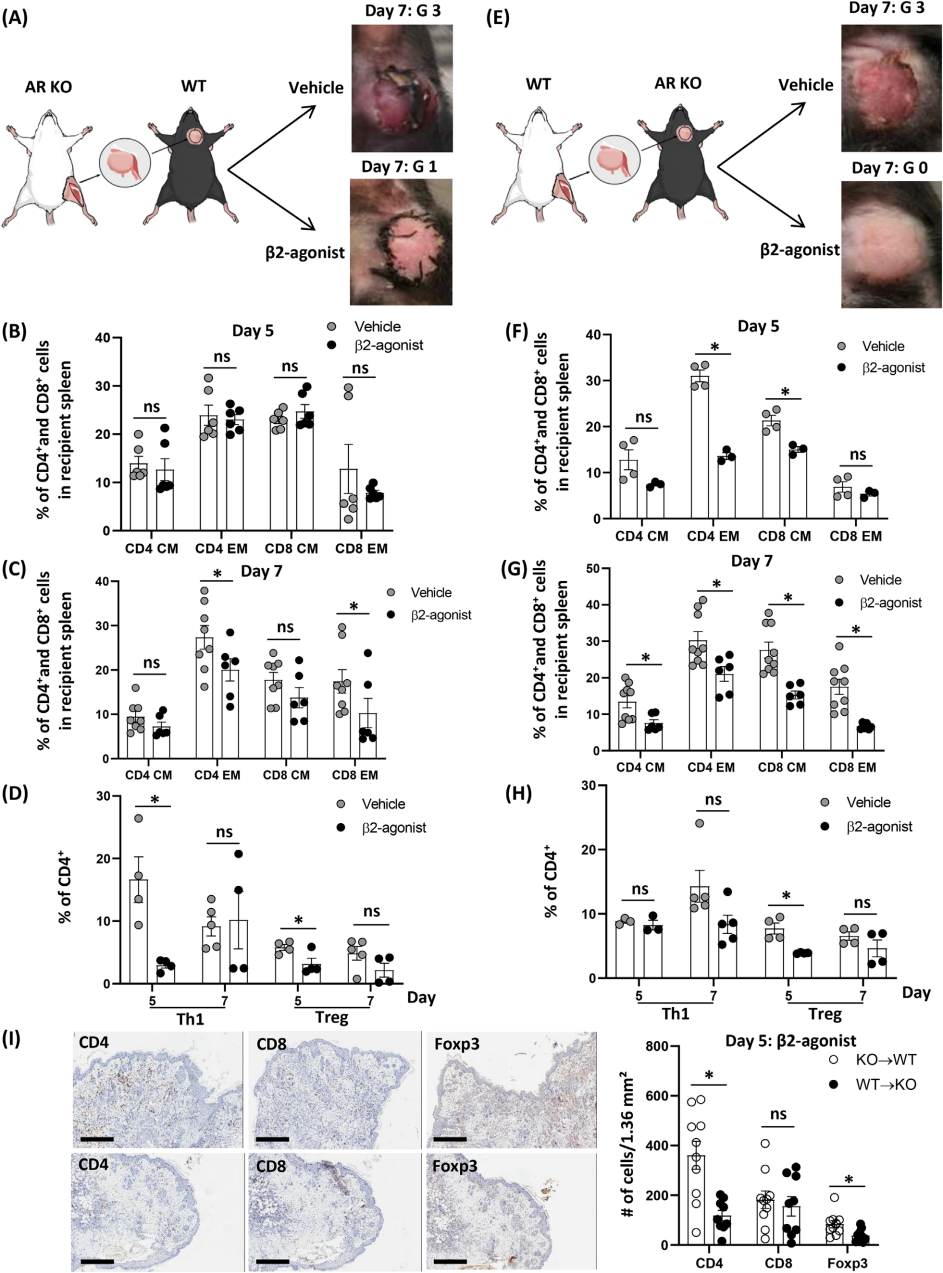
Modulation of β_2_‐adrenergic receptor (AR) signalling in donors is more effective to delay rejection responses than the modulation in recipients. (A) BALB/c β_2_‐AR knock‐out (KO) donors were transplanted to C57BL/6 recipients with either vehicle or β_2_‐agonist injections. (B and C) Compositions of CD4^+^/CD8^+^ central memory (CM)/effector memory (EM) T‐cell populations in the spleen 5 and 7 days with either vehicle or β_2_‐agonist after vascularized composite tissue allotransplantation (VCA) without tacrolimus in wild‐type (WT) recipients receiving BALB/c β_2_‐AR KO grafts. Day 5, *n* = 6; day 7, *n* ≥ 6. ns, not significant; error bar, standard error of the mean. *p < .05 by Student's *t* test. (D) Th1‐ and Treg‐cell populations in CD4^+^ T cells after vehicle or β_2_‐agonist treatments in WT recipients receiving BALB/c β_2_‐AR KO grafts. *n* ≥ 4. ns, not significant; error bar, standard error of the mean. *p < .05 by Student's *t* test. (E) BALB/c WT donors were transplanted to C57BL/6 β_2_‐AR KO recipients with either vehicle or β_2_‐agonist injections. (F and G) Compositions of CD4^+^/CD8^+^ CM/EM T‐cell populations in the spleen 5 and 7 days with either vehicle or β_2_‐agonist after VCA without tacrolimus in β_2_‐AR KO recipients receiving BALB/c WT grafts. Day 5, *n* ≥ 3; day 7, *n* ≥ 6. ns, not significant; error bar, standard error of the mean. *p < .05 by Student's *t* test. (H) Th1‐ and Treg‐cell populations in CD4^+^ T cells with either vehicle or β_2_‐agonist treatments in β_2_‐AR KO recipients receiving BALB/c WT grafts. *n* ≥ 3. ns, not significant; error bar; standard error of the mean. *p < .05 by Student's *t* test. (I) Representative figures for immunohistochemistry (IHC) with CD4, CD8 and Foxp3 antibodies with β_2_‐agonist treatment for 5 days in WT recipients bearing AR KO donor's grafts (KO → WT) and AR KO recipients bearing WT donor's grafts (WT → KO). Over nine fields from *n* = 3/group. ns, not significant; error bar, standard error of the mean; scale bar: 400 µm. *p < .05 by Student's *t* test

**FIGURE 7:**
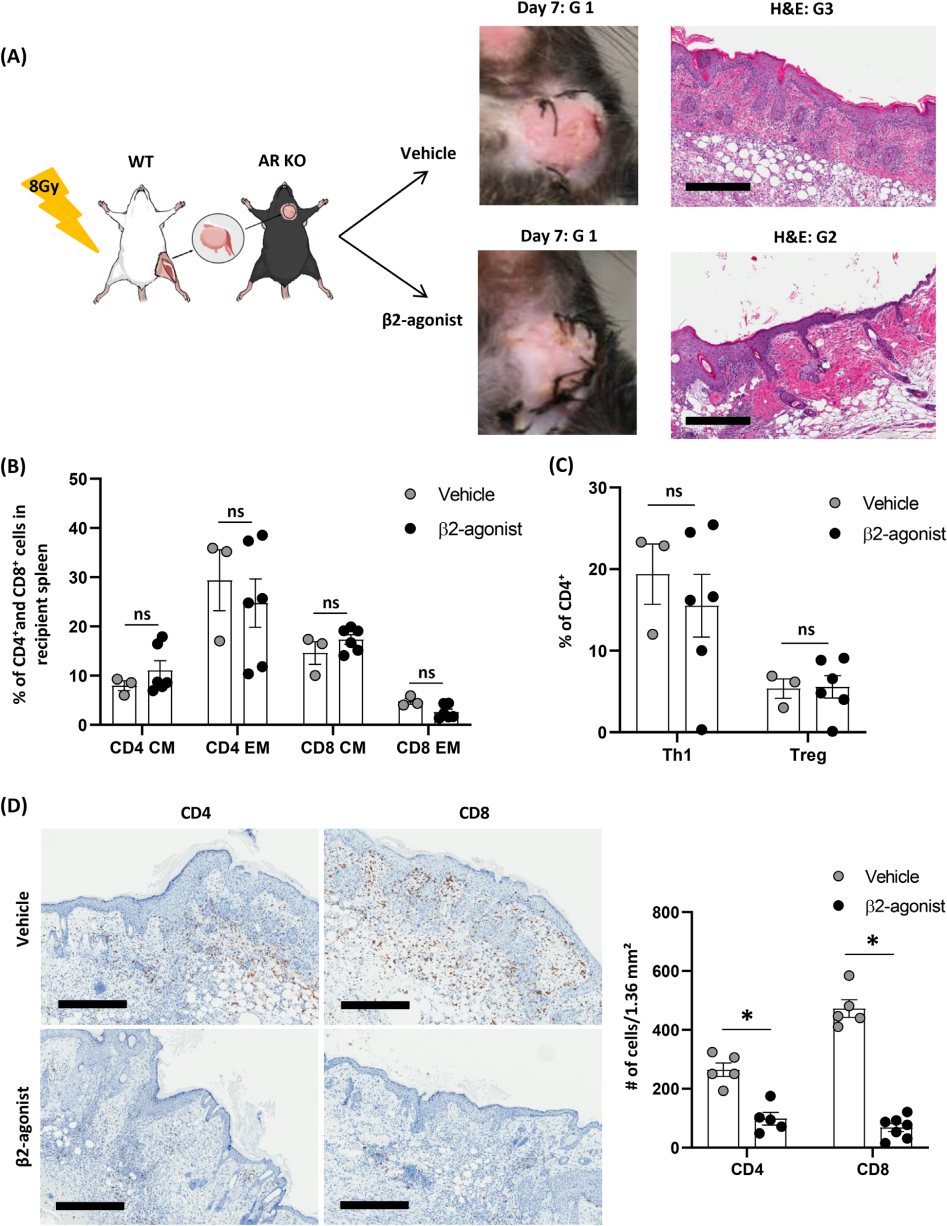
β_2_‐Adrenergic receptor (AR) agonist has effects with donor's stromal cells to inhibit T‐cell trafficking into transplanted grafts. (A) BALB/c wild‐type (WT) mice as a donor were exposed to 8‐Gy irradiation the day before surgery, the composite graft was transplanted to C57BL/6 β_2_‐AR knock‐out (KO) recipients with either vehicle or β_2_‐agonist treatments without tacrolimus. (B) Compositions of CD4^+^/CD8^+^ CM and (C) effector memory (EM) T‐cell populations and Th1 and Treg populations. *n* ≥ 3. ns, not significant; error bar, standard error of the mean. *p < .05 by Student's *t* test. (D) Immunohistochemistry (IHC) with CD4/CD8 antibodies, and cells were counted by ImageJ programme. Over five fields from *n* = 3/group. ns, not significant; error bar, standard error of the mean; scale bar: 400 µm. *p < .05 by Student's *t* test

β_2_‐AR normally exists in both donors and recipients after transplant, so it was necessary to investigate whether β_2_‐agonist activated AR signals on donors’ or recipients’ cells to delay rejection responses. Well‐studied AR KO mouse strains as donors and recipients were used to investigate the specific role of β_2_‐AR in the treatments of either donor or recipient.^34^ Grafts were harvested from BALB/c AR KO mice as donors, and transplanted into C57BL/6 WT recipients (Figure 6A–D). Although the graft showed less severe gross rejection as grade 1 with β_2_‐agonist compared to the vehicle injection (Figure 6A), no statistical difference was found in the compositions of CD4^+^ and CD8^+^ memory T‐cell populations at day 5 in the spleen (Figure 6B). However, the composition of CD4^+^ and CD8^+^ EM T‐cell populations were significantly lower in the β_2_‐agonist group than the vehicle group at day 7 (Figure 6C), and significantly less Th1 and Treg were detected with β_2_‐agonist compared to vehicle at day 5 (Figure 6D). In parallel, WT grafts were transplanted to C57BL/6 AR KO recipients with either vehicle or β_2_‐agonist injections (Figure 6E‐H). Significant decreases in CD4^+^ and CD8^+^ memory T‐cell populations were detected at day 5 (Figure 6F) and 7 (Figure 6G) with gross grade 0 rejection in the β_2_‐agonist‐treated group compared to the vehicle group (grade 3). In addition, β_2_‐agonist decreased Treg population significantly compared to vehicle injection at day 5 (Figure 6H). Furthermore, significant decreases in CD4^+^ T cell and Treg infiltration were detected with β_2_‐agonist treatment in AR KO recipients bearing the WT grafts (WT → KO) compared to WT recipients bearing AR KO grafts (KO → WT) (Figure 6I). Although the infiltrated lymphocytes in grafts originated from recipients (Figure S3), the manipulation of donor's β_2_‐AR signals is critical for suppressing T‐cell infiltration and rejection responses.

### 3.7 Important actions of β_2_‐AR agonist on donor stromal cells after VCA

**FIGURE 8:**
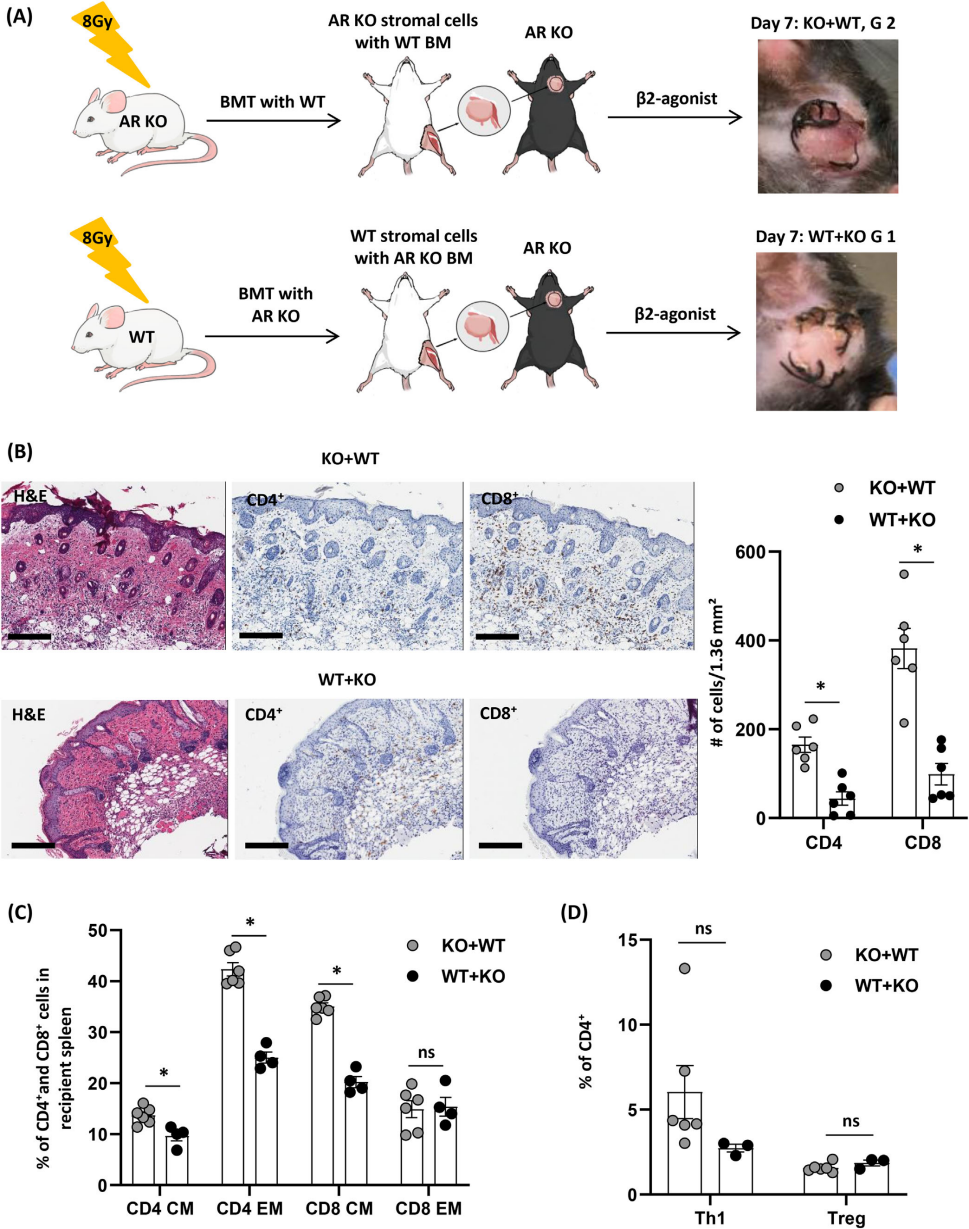
β_2_‐Adrenergic receptor (AR) agonist has effects with donor's stromal cells to inhibit T‐cell trafficking into transplanted grafts. (A) BALB/c AR knock‐out (KO) and wild‐type (WT) donors were exposed to 8 Gy irradiation, and then BALB/c WT and AR KO bone marrow (BM) were transplanted respectively. After 8‐week bone marrow transplantation (BMT), vascularized composite tissue allotransplantation (VCA) was performed on C57BL/6 AR KO recipients followed by β_2_‐agonist treatment for 7 days. (B) Haematoxylin and eosin (H&E) and immunohistochemistry (IHC) with CD4 and CD8 antibodies, six fields from *n* = 3/group. Error bar, standard error of the mean; scale bar: 200 µm. *p < .05 by Student's *t* test. (C) Systemic compositions of CD4^+^/CD8^+^ central memory (CM)/effector memory (EM) T‐cell populations and (D) Th1‐ and Treg‐cell populations in CD4^+^ T cells 7 days after VCA with β_2_‐agonist treatment in C57BL/6 AR KO recipients having a graft from either BALB/c β_2_‐AR KO with WT BMT (KO + WT) or BALB/c WT with AR KO BMT (WT + KO). *n* ≥ 3. ns, not significant; error bar, standard error of the mean. *p < .05 by Student's *t* test

Since our VCA model contains a donor's femur with BM, it was important to discriminate the effects of β_2_‐agonist on hematopoietic and stromal cells in transplanted grafts. β_2_‐AR modulation in donors suppresses rejection responses after VCA, so to better determine the responsible populations of cells, BM and stromal cells of the donor were examined. WT donors were exposed to 8‐Gy irradiation the day before VCA to eradicate BM cells, and then grafts, including a femur, were transplanted to β_2_‐AR KO recipients followed by vehicle or β_2_‐agonist injections. Both groups showed mild xerosis of grafts with intact skin anatomy (Figure 7A), and distinct effects of β_2_‐agonist treatment were lost in the CD4^+^ and CD8^+^ memory T cell (Figure 7B), Th1 and Treg (Figure 7C) populations 7 days after VCA. However, significantly lower numbers of CD4^+^ and CD8^+^ T‐cell infiltrations were found in the β_2_‐agonist‐treated graft than the vehicle group (Figure 7D). Further, a β_2_‐AR KO donor was irradiated, and then WT BMT was performed to generate a chimeric model, including β_2_‐AR KO stromal cells and WT BM, and vice versa (Figure 8A). Grafts composed of β_2_‐AR KO stromal with WT BM (KO + WT) showed more severe rejection than WT stromal with AR KO BM (WT + KO) grafts in AR KO recipients (Figure 8A,B; KO + WT gross grade 2 and histologic grade 3, WT + KO gross grade 1 and histologic grade 2). Greater amounts of CD4^+^ T and CD8^+^ T‐cell infiltration were found in the KO + WT grafts than the WT + KO grafts (Figure 8B). Additionally, significant decreases of CD4^+^ and CD8^+^ memory T‐cell populations were detected in the systemic immune responses of mice bearing WT + KO grafts compared to mice bearing KO + WT grafts (Figure 8C) with no significant difference on Th1 and Treg populations between groups (Figure 8D). It means that β_2_‐AR signals in donor's stromal cells have critical roles on recipient's T‐cell trafficking.

### 3.8 Inhibition of endothelial adhesion molecules and T‐cell trafficking chemokines with β_2_‐AR agonist

**FIGURE 9:**
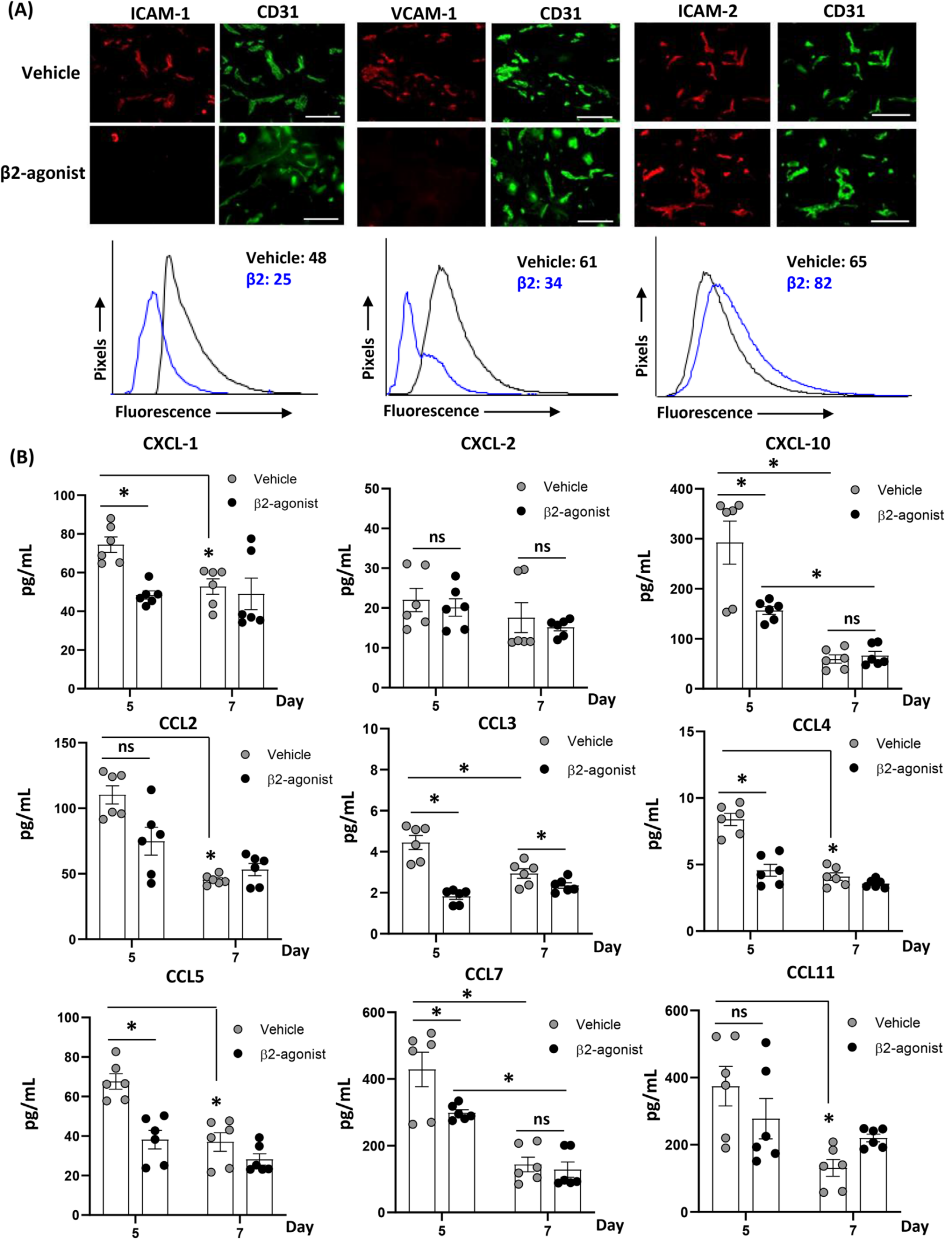
β_2_‐Adrenergic receptor (AR) agonist suppresses intercellular adhesion molecule‐1 (ICAM‐1) and vascular cell adhesion molecule‐1 (VCAM‐1) expression in donor grafts and significantly decreases CXCL‐1, CCL4 and CCL5 in recipients’ blood. (A) Immunofluorescence (IF) and image quantification were performed 5 days after vascularized composite tissue allotransplantation (VCA) with wild‐type (WT) donors and recipients followed by either vehicle or β_2_‐agonist injections. ICAM‐1, VCAM‐1, ICAM‐2 and CD31 antibodies were used. Histograms depict quantification of IF intensity of each antibody in all CD31^+^ vessels; numbers denote micro‐flow imaging (MFI). All data are representative of duplicate experiments, *n* = 3; scale bar: 100 µm. (B) Using Luminex assay, in the setting of WT donors and WT recipients VCA without tacrolimus, various chemokine levels were analysed 5 and 7 days after transplant. *n* = 6; pooled data from duplicate samples. ns, not significant; error bar, standard error of the mean. *p < .05 by Student's *t* test

Multiple mechanisms and steps are required for leukocytes to infiltrate into stromal tissue of the donor's graft, and we wondered whether β_2_‐agonist could change any of these mechanisms. Leukocyte extravasation is one of the essential and first steps during the initiation of cell‐mediated rejection, and ICAM‐1/2 and VCAM‐1 are the endothelial adhesion molecules that mediate firm adhesion just prior to cell extravasation.^46^ In addition, chemokines play a central role in directing the migration of leukocytes.^47^ To investigate changes of endothelial adhesion molecules in donor's vessels after β_2_‐agonist treatment, WT donor's grafts in WT recipients were stained 5 days after VCA. β_2_‐Agonist suppressed ICAM‐1 and VCAM‐1 expression in graft vessels compared to vehicle, whereas the expression of ICAM‐2 was increased in the β_2_‐agonist‐treated group (Figure 9A) by immunofluorescence. Also, recipient's plasma levels of the relevant T‐cell trafficking chemokines CXCL‐1, CXCL‐10, CCL2, CCL3, CCL4, CCL5, CCL7 and CCL11 increased significantly at day 5, and resolving by day 7. Under β_2_‐agonist treatment, levels of CXCL‐1, CXCL‐10, CCL3, CCL4, CCL5 and CCL7, which are pro‐inflammatory chemokines that regulates leukocytes trafficking,^48^ were decreased significantly compared to the vehicle injection (Figure 9B). This was further supported by the time and treatment interactions in two‐way ANOVA except for CXCL‐2, which suggests that the changes in these chemokine levels are significantly different between treatment groups over time (Table S3). This finding suggests that β_2_‐agonist manipulated expression levels of endothelial adhesion molecules in donor's stromal cells and suppressed the production of numerous chemokines which were capable of leukocyte trafficking in recipients.

### 3.9 Effects of donor preconditioning prior to VCA with β_2_‐AR agonist

If the same effects of rejection responses are achieved with β_2_‐agonist treatment in a donor prior to VCA, the application of β_2_‐agonist can be extended to donors. β_2_‐Agonist treatment before VCA was examined as a pre‐conditioning regimen as BALB/c WT mice were given β_2_‐agonist for 2 weeks prior to VCA (pre‐VCA) and compared to the previous experiments injecting β_2_‐agonist after VCA (post‐VCA) (Figure S9A). Although similar gross and histologic rejection grades were detected in the pre‐VCA group (grades 1 and 3 respectively) compared to the post‐VCA group (grades 0 and 3 respectively) 7 days after surgery, the immunosuppressive effects on memory T‐cell populations in recipients were lost in the setting of pre‐VCA (Figure S9B). But the suppression of the Th1 population and CD8^+^ T‐cell trafficking in pre‐VCA were comparable to post‐VCA (Figure S9C,D). This experiment reveals that the preconditioning with β_2_‐AR agonist in donors achieves partial effects of immunosuppression compared to the usage in recipients.

## 4 DISCUSSION

Vascularized composite allographs are composed of multiple tissues with different immunogenic and functional properties, including skin, muscles, bones and nerves, and they are typically obtained from cadavers. Thus, there is little chance to match HLA types with the recipients. As a result, these donor tissues are highly antigenic requiring very large and toxic doses of drugs such as tacrolimus.^13^, ^49^, ^50^ Here, we tested a strategy that mimics the natural ability of nerves and AR signalling by catecholamines such as NE to suppress anti‐graft T‐cell‐mediated immune responses and prolong survival of a complex tissue allograft. Mimicking the natural (and temporary) suppressive function of nerves on the immune response to promote survival of VCA (or even that of more traditional solid organ transplants) has not previously been tested in either preclinical or clinical settings.

Several groups have tested minimizing the use of immunosuppressants (tacrolimus or steroids) after VCA relying instead on the benefits of steroid‐free immunosuppression in solid organ transplants; unfortunately, this approach has been associated with frequent acute rejection episodes.^51^, ^52^ Importantly, we found that we could reduce the dose of a standard immunosuppressant drug tacrolimus to half and improve graft survival by augmenting low‐dose tacrolimus with a β_2_‐AR agonist. It the future, it may be possible that β_2_‐AR agonists could replace steroids to prevent development of steroid‐related complications, but this remains to be tested. Selective β_2_‐AR targeting drugs have been safely and extensively used for decades for other indications, particularly for cardiovascular and pulmonary manipulation such as in cases of asthma.^53^ In studies on asthma in humans, researchers have even safely used higher doses of terbutaline compared to the recommended dose needed to diminish asthmatic symptoms.^54^ At the dose of terbutaline that we used here to prolong graft survival, no changes in HR and BP were observed with daily use, and no drug interaction with tacrolimus was found, thus supporting the safety of this approach.

Mice have a more rapid metabolism than humans,^55^ and therefore it is not always possible to draw a direct comparison between the effects of various pharmacological drugs, nor to extrapolate optimal doses from mouse to human. For example, dopamine has been prescribed to treat low BPs in patients. But, low infusion rates act on the visceral vessels to produce vasodilation resulting in increased urinary flow; on the other hand, higher doses cause vasoconstriction and increased BP via the ARs α_1_, β_1_ and β_2_.^56^ For these reasons, dose escalation studies of β_2_‐agonists are needed to optimize future combination regimens with tacrolimus in patients.

Without treatment with β_2_‐AR agonist, we observed here that CD4^+^ T and CD8^+^ T cells were increased significantly in transplanted grafts at the time of rejection. β_2_‐AR agonist treatment was associated with significantly fewer numbers of infiltrated T cell and decreased EM T and Th1 cell populations in recipients corresponding to the prolonged survival benefit. In addition, we have previously shown that treating stressed mice with β‐AR *antagonists* alleviated mitochondrial dysfunction, increased glycolysis in CD8^+^ T cells and increased T‐cell activation resulting in reduced tumour growth rates and significantly fewer exhausted T cells.^57^ It is likely that β‐AR *agonists* may have inhibitory effects on T‐cell activation, and we observed that β‐agonists impaired T‐cell receptor signalling,^57^ thus reducing their graft destruction properties. Although several clinical and preclinical studies have shown that Tregs modulate immune responses and that a high level of Treg populations are predictive for a better prognosis after solid organ and haematopoietic cell (HPC) transplantation,^58^ in our VCA model increased numbers of Tregs were detected in the presence of strong immune responses after transplant. Thus, recipients with greater levels of Treg populations showed severe gross rejection at early time points and eventual resolution of the population in the recipient's blood and grafts after severe gross rejection was well underway. Although we do not, as yet, understand the basis of this difference, it may be related to the highly antigenic VCA grafts, which contain regions of skin, as opposed to those used in solid organ transplants, particularly at early time points. Organ transplantations have also been associated with increased numbers of immunosuppressive MDSC,^59^, ^60^, ^61^ and as the MDSC population will likely increase with β_2_‐agonist treatment after VCA (based on β_2_‐agonist effects in the setting of GVHD after BMT^34^), these cells may be a more potent contributor to immunosuppression than Treg induction.

In terms of the immune modulatory impact of adding a β_2_‐AR agonist, terbutaline alone delayed rejection responses in association with significant decreases in CD4^+^/CD8^+^ EM T‐ and Th1‐cell populations, cytokines and chemokines such as IFN‐γ, IL‐6, TNF‐α, CXCL‐1/10 and CCL3/4/5/7 in recipients. Addition of the β_2_‐AR agonist treatment increased the anti‐inflammatory cytokine, IL‐13, which was associated with concomitant down‐regulation of TNF‐α production, a phenomenon observed in other studies.^62^, ^63^ We found that IL‐18, CCL2 and CCL11 were particularly unresponsive to β_2_‐agonist treatment indicating that these cytokine/chemokines might be targetable by other agents to further suppress rejection responses after VCA.

β_2_‐AR treatment of either recipients or donors was sufficient to delay gross rejection responses, but treatment of the donor resulted in a more potent effect. Specifically, β_2_‐AR treatment of the donor tissue stromal cells was necessary to suppress recipient T‐cell trafficking into grafts. Leukocyte extravasation is a prerequisite for acute rejection in grafts, and their migration into the tissue requires the expression of adhesion molecules on the surface of activated endothelium.^64^, ^65^ The endothelial adhesion molecules ICAM‐1 and VCAM‐1 are known as the central mediators of leukocyte adhesion to and transmigration across the endothelium,^46^ and β_2_‐AR agonist suppressed ICAM‐1 and VCAM‐1 expression with concomitant decreased T‐cell trafficking into grafts. Of note, ICAM‐2 expression was increased. As previously reported, during longer periods of inflammation, endothelial cells respond to inflammatory mediators by massive up‐regulation of adhesion receptors such as ICAM‐1 and VCAM‐1, whereas ICAM‐2 decreases.^66^, ^67^, ^68^ A possible explanation for our observed increase in ICAM‐2 is that it might represent a compensatory mechanism for decreased ICAM‐1 or an additional effect of β_2_‐AR modulation. The β_2_‐AR agonist, terbutaline stimulated angiogenesis on endothelial cells derived from the central nervous system,^69^ and ICAM‐2 has been associated with this process.^70^ By regulating ICAM‐2 and increasing angiogenesis, β_2_‐agonist treatment could reduce ischemic injury in harvested organs. Graft salvage effects of β_2_‐agonist suggest that regulation of endothelial adhesion molecules partially contributes for the prevention of chronic rejection after VCA (Figure 4). Another possible mechanism underlying benefits of a β_2_‐agonist is that it can increase cAMP (cyclic adenosine monophosphate) levels^71^; cAMP is known as a potent negative regulator in T cells, which dampens T‐cell‐immune function through the cAMP/protein kinase A signalling pathway.^72^

β_2_‐AR activation in donor BM cells was necessary for the observed immunosuppressive effects, including suppression of IFN‐γ levels in recipients. Although the donor's HPCs were replaced by the recipient's HPC within 5 days after VCA in our model (Figure S2), it is likely that a transient mixed chimerism developed in the recipients, which was sufficient for the induction of allograft tolerance.^73^, ^74^, ^75^ Although the study of transplant tolerance is beyond the scope of this study, β_2_‐agonist treatment could be a mechanism to facilitate the induction of tolerance and could replace at least some of the more toxic immunosuppressive drugs currently being used.

Interestingly, in a previous study, using allografts from cadaveric kidney donors who had been treated prior to death with dopamine and NE to maintain their BP and HR resulted in reduced acute rejection and improved graft survival after transplantation.^76^, ^77^ Our current findings suggest a possible mechanism for this observation. β_2_‐Agonist addition to preservation solutions may be a relatively simple measure to improve organ transplantation, in addition to the treatment of recipients.

One problem with our approach is that terbutaline is a relatively short acting β_2_‐agonist. It is likely that longer acting agonists (e.g. bambuterol and salmeterol) will be superior to terbutaline, and this assumption is supported by our experiment with bambuterol, a long‐acting β_2_‐agonist (Figure 5). Another issue is that among our experiments, we noticed some differences in immune subpopulations of animals given the same treatment modality (e.g. Figure 3A,D). Even though the animals were purchased from the same vendor, perhaps small variations in housing factors, or physiological differences in the status of the microbiome, could be a reason for this variation. Published data demonstrate that changes in microbiota may cause different rejection responses after skin transplant.^78^ Another limitation is that our study is focused largely upon effector T lymphocytes and their role in graft rejection. Even though current immunosuppressive therapies for solid organ transplantation appear to be dominated by T‐cell mechanisms, β_2_‐AR exists on B lymphocytes, and innate immune cells such as granulocytes, macrophages, dendritic cells and natural killer cells.^79^, ^80^ Thus, other immune cell subtypes, which are also associated with graft rejection, may contribute to some of our observed findings, and this should be addressed in future studies. Finally, it will be important to determine whether β_2_‐agonists can enhance lower doses of other conventional immunosuppressive drugs such as glucocorticoids, alkylating agents, and purine synthesis inhibitors.

## 5 CONCLUSIONS

There is a significant need to improve graft survival and reduce the doses of toxic immunosuppressant drugs currently being given to patients receiving VCA. We have demonstrated here that by pharmacologically targeting adrenergic stress pathways ordinarily used physiologically by nerves of the autonomic nervous system, we could not only prolong graft survival but more importantly, allow for reduction of the dose of a standard immunosuppressive drug used in various VCA scenarios. We have also identified several relevant mechanisms by which this prolongation of the survival of VCA occurs. Future studies will certainly need to optimize the dose and scheduling of applications of β‐agonists and test additional combinations with immunosuppressive drugs so that the best protocols can be offered to patients receiving VCA in the near future.

## CONFLICT OF INTEREST

The authors reported no proprietary or commercial interest in any product mentioned or concept discussed in the article.

## Supporting information

FIGURE S1 Gating strategies for flow cytometry

FIGURE S2 A schematic illustration of our vascularized composite tissue allotransplantation (VCA) model and the safety of a selective β_2_‐adrenergic receptor (AR) agonist drug, terbutaline. (A) BALB/c and C57BL/6 strains were used as donors and recipients respectively. En bloc tissue composed of skin, subcutaneous fat, muscle, vessels and femur was transplanted to a recipient's cervical area. (B) Systolic and diastolic blood pressures (BP) with heart rates (HRs) were measured after everyday injection of with either vehicle (V; PBS) or β_2_‐agonist (β; terbutaline; 2 mg/day). Mice were acclimated to the BP and HR measuring procedures for 10 days before recording data. Representative data between two different experiments, *n* = 5. (C) Concentrations of tacrolimus in plasma were analysed 14 days after subcutaneous injections (24 h after the last injection) with either a half dose of tacrolimus (hTac; 2 mg/kg/day) or a full dose of tacrolimus (fTac; 4 mg/kg/day). Representative data between two different experiments, *n* = 5. ns, not significant; error bar, standard error of the mean. *p < .05 by Student's *t* test

FIGURE S3 Pathologic findings 5 and 7 days after VCA with either vehicle or β_2_‐agonist injections. (A and B) Representative haematoxylin and eosin (H&E) images revealed epithelial dyskeratosis (arrowhead) and apoptosis (arrow) in the vehicle‐injected group (A; rejection grade 3) not in the β_2_‐agonist‐injected group (B; rejection grade 2) 5 days after VCA, scale bar: 50 µm. (C and D) Representative figures for H&E and immunohistochemistry (IHC) with CD8, CD4 and Foxp3 antibodies either with vehicle or β_2_‐agonist treatment for 7 days. (E) Numbers of CD4, CD8 and Foxp3 positive cells in grafts 7 days after VCA. Nine fields from three grafts per group. ns, not significant; error bar, standard error of the mean. (F) Compositions of CD4^+^/CD8^+^ central memory (CM) and effector memory (EM) T‐cell populations in transplanted grafts 7 days after VCA. Control, non‐vascularized grafts; *n* = 5. ns, not significant; error bar, standard error of the mean; scale bar: 400 µm. *p < .05 by Student's *t* test

FIGURE S4 Pathology findings of CD4 T‐cell infiltration 5 days after VCA. Representative IHC images of nine different transplanted grafts revealed CD4 T‐cell infiltration in the vehicle (A) and β_2_‐agonist (B) injected groups, scale bar: 400 µm

FIGURE S5 Pathology findings of CD8 T‐cell infiltration 5 days after VCA. Representative IHC images of nine different transplanted grafts revealed CD8 T‐cell infiltration in the vehicle (A) and β2‐agonist (B) injected groups, scale bar: 400 µm

FIGURE S6 Pathology findings of Foxp3 cell infiltration 5 days after VCA. Representative IHC images of nine different transplanted grafts revealed Foxp3 cell infiltration in the vehicle (A) and β2‐agonist (B) injected groups, scale bar: 400 µm

FIGURE S7 The composition of donor's and recipient's leukocytes in donor grafts and recipient blood 5 and 7 days after VCA. (A and B) Source of infiltrated T cells in transplanted grafts and recipient's blood between recipient (CD45‐1) and donor (CD45‐2). Control, non‐vascularized grafts; *n* ≥ 3. ns, not significant; error bar, standard error of the mean. *p < .05 by Student's *t* test

FIGURE S8 The correlation of T‐cell infiltration and numbers of Foxp3^+^ cell in transplanted grafts after VCA. (A) Correlation analysis between numbers of infiltrated CD4/8 T and Foxp3^+^ cells in transplanted grafts (*n* = 8) 5 days after vehicle injections. (B) Correlation analysis between numbers of infiltrated CD4/8 T and Foxp3^+^ cells in transplanted grafts (*n* = 8) 5 days after β_2_‐agonist injections. The composition of Treg (CD4^+^CD25^+^Foxp3^+^) population was analysed with fTac injections after VCA. (C) Representative figure for H&E and IHC with numbers of CD4, CD8 and Foxp3 cells 10 days after VCA. Five fields from three grafts. Error bar, standard error of the mean; scale bar: 400 µm. (D) The Treg population was analysed with recipient's spleen 30 days after VCA. Control, a mouse without VCA; *n* = 4. ns, not significant; error bar, standard error of the mean

FIGURE S9 Preconditioning in donors with β_2_‐AR agonist delays rejection responses through suppression of T‐cell trafficking in the grafts. (A) BALB/c donor mice were injected with β_2_‐agonist for 2 weeks before VCA (pre‐VCA), and then β_2_‐agonist treatment was stopped after the surgery in C57BL/6 AR KO recipients. Representative figures, scale bar: 400 µm. (B) Systemic compositions of CD4^+^/CD8^+^ CM and EM T‐cell populations 7 days after VCA. (C) Th1‐ and Treg‐cell populations in CD4^+^ T cells. *n* ≥ 4 mice. ns, not significant; error bar, standard error of the mean. *p < .05 by Student's *t* test. (D) Numbers of CD4 and CD8 positive cells in grafts 7 days after VCA. Over 10 fields from 3 mice/group. ns, not significant; error bar, standard error of the mean; scale bar: 400 µm. Data with empty circles (post‐VCA); historical data. *p < .05 by Student's *t* test

TABLE S1 Two‐way ANOVA analysis in the number of infiltrating CD4^+^ T and CD8^+^ T cells from day 5 to 7
TABLE S2 Two‐way ANOVA analysis in changes of cytokine levels from day 5 to 7
TABLE S3 Two‐way ANOVA analysis in changes of chemokine levels from day 5 to 7

Supporting Information
