# Supplementary figures and images for: Manipulating adrenergic stress receptor signalling to enhance immunosuppression and prolong survival of vascularized composite tissue transplants
Source: Clin Transl Med. 2022 Aug 22;12(8):e996. doi: 10.1002/ctm2.996 (PMC9394753; doi:10.1002/ctm2.996)

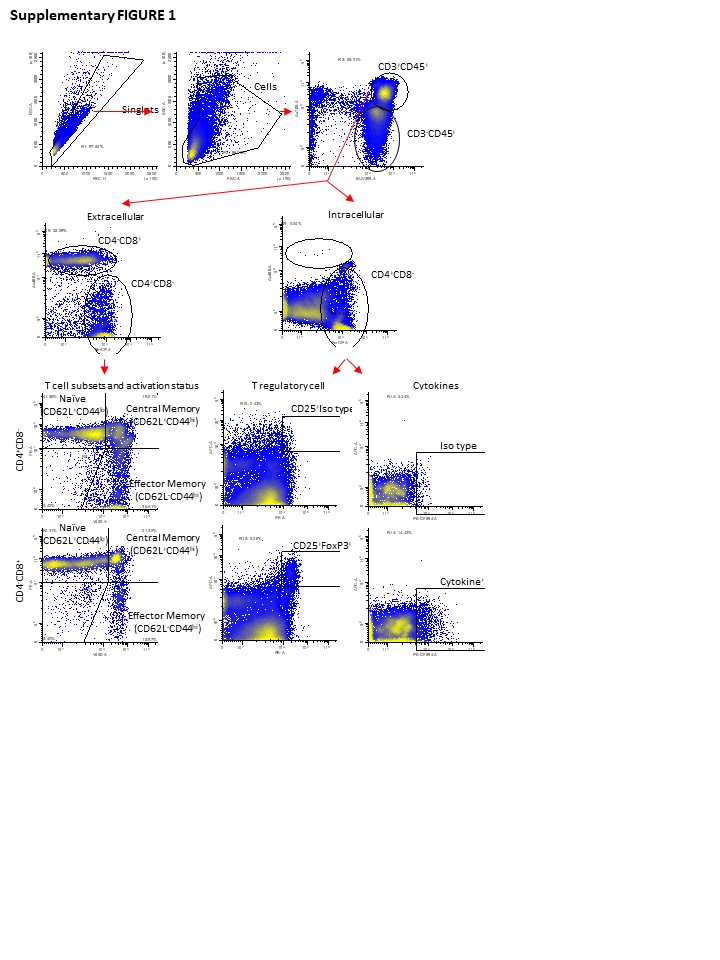

Supplement: Supplementary file 1 — FIGURE S1 Gating strategies for flow cytometry [file CTM2-12-e996-s003.JPG]

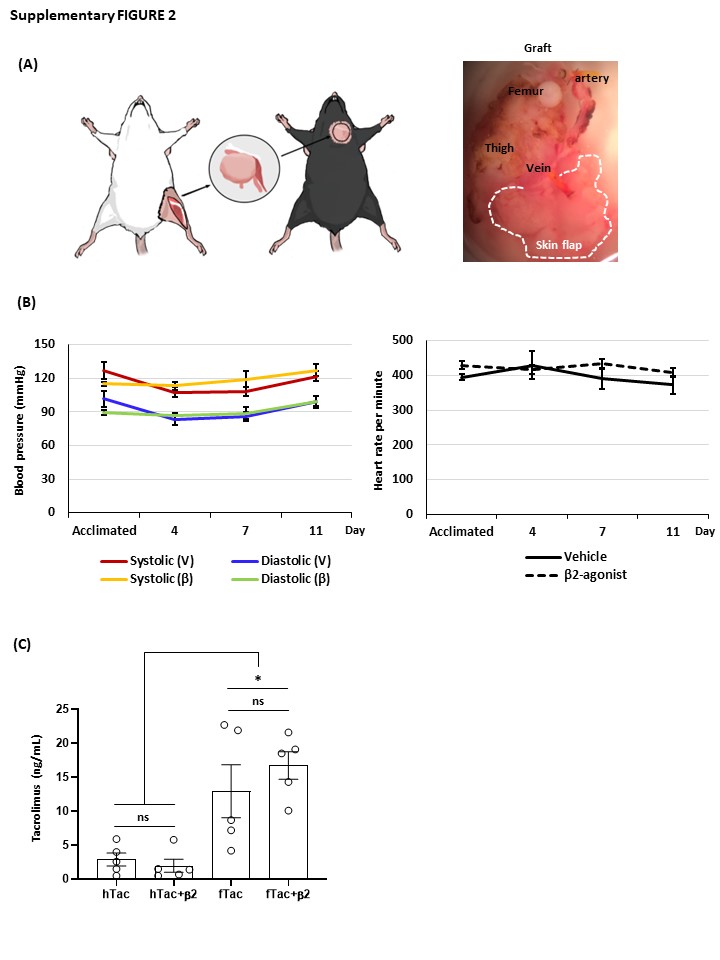

Supplement: Supplementary file 2 — FIGURE S2 A schematic illustration of our vascularized composite tissue allotransplantation (VCA) model and the safety of a selective β2‐adrenergic receptor (AR) agonist drug, terbutaline. (A) BALB/c and C57BL/6 strains were used as donors and recipients respectively. En bloc tissue composed of skin, subcutaneous fat, muscle, vessels and femur was transplanted to a recipient's cervical area. (B) Systolic and diastolic blood pressures (BP) with heart rates (HRs) were measured after everyday injection of with either vehicle (V; PBS) or β2‐agonist (β; terbutaline; 2 mg/day). Mice were acclimated to the BP and HR measuring procedures for 10 days before recording data. Representative data between two different experiments, n = 5. (C) Concentrations of tacrolimus in plasma were analysed 14 days after subcutaneous injections (24 h after the last injection) with either a half dose of tacrolimus (hTac; 2 mg/kg/day) or a full dose of tacrolimus (fTac; 4 mg/kg/day). Representative data between two different experiments, n = 5. ns, not significant; error bar, standard error of the mean. *p < .05 by Student's t test [file CTM2-12-e996-s008.JPG]

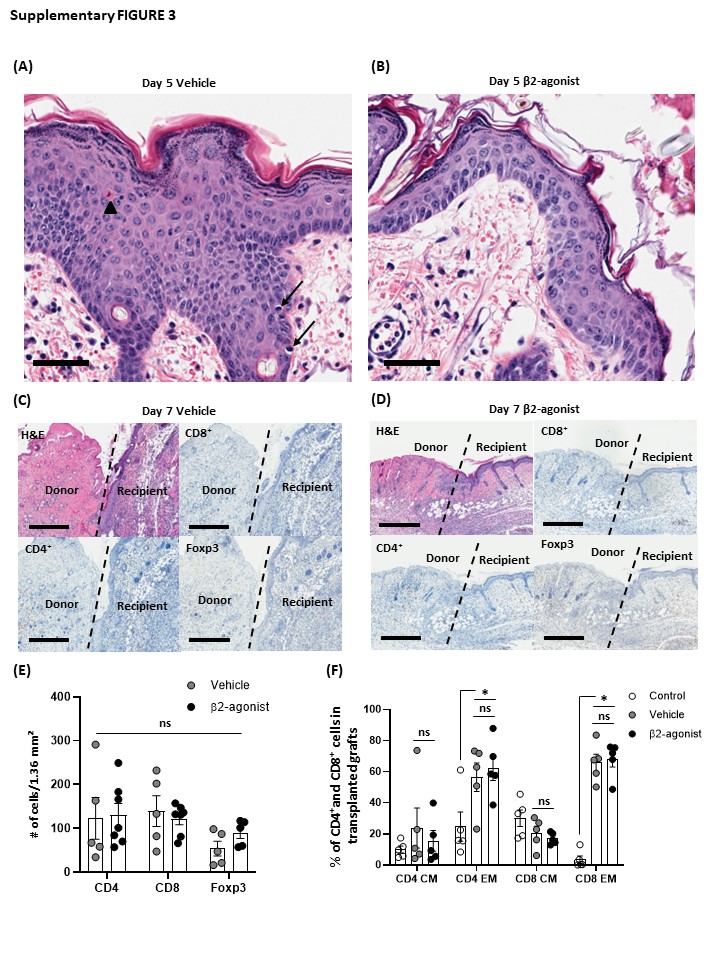

Supplement: Supplementary file 3 — FIGURE S3 Pathologic findings 5 and 7 days after VCA with either vehicle or β2‐agonist injections. (A and B) Representative haematoxylin and eosin (H&E) images revealed epithelial dyskeratosis (arrowhead) and apoptosis (arrow) in the vehicle‐injected group (A; rejection grade 3) not in the β2‐agonist‐injected group (B; rejection grade 2) 5 days after VCA, scale bar: 50 µm. (C and D) Representative figures for H&E and immunohistochemistry (IHC) with CD8, CD4 and Foxp3 antibodies either with vehicle or β2‐agonist treatment for 7 days. (E) Numbers of CD4, CD8 and Foxp3 positive cells in grafts 7 days after VCA. Nine fields from three grafts per group. ns, not significant; error bar, standard error of the mean. (F) Compositions of CD4+/CD8+ central memory (CM) and effector memory (EM) T‐cell populations in transplanted grafts 7 days after VCA. Control, non‐vascularized grafts; n = 5. ns, not significant; error bar, standard error of the mean; scale bar: 400 µm. *p < .05 by Student's t test [file CTM2-12-e996-s005.JPG]

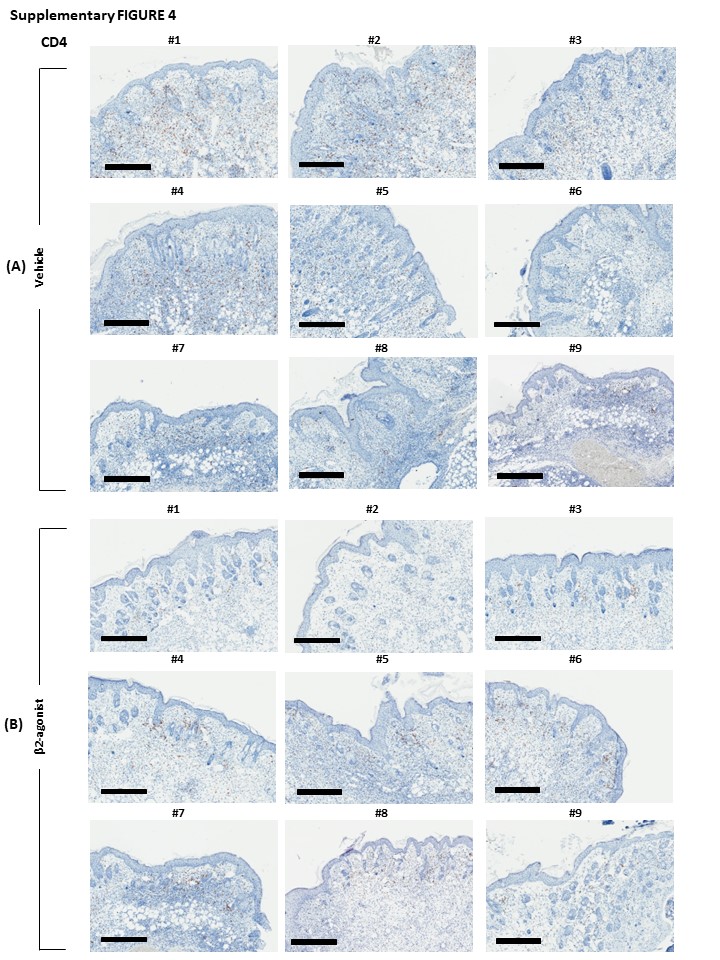

Supplement: Supplementary file 4 — FIGURE S4 Pathology findings of CD4 T‐cell infiltration 5 days after VCA. Representative IHC images of nine different transplanted grafts revealed CD4 T‐cell infiltration in the vehicle (A) and β2‐agonist (B) injected groups, scale bar: 400 µm [file CTM2-12-e996-s009.JPG]

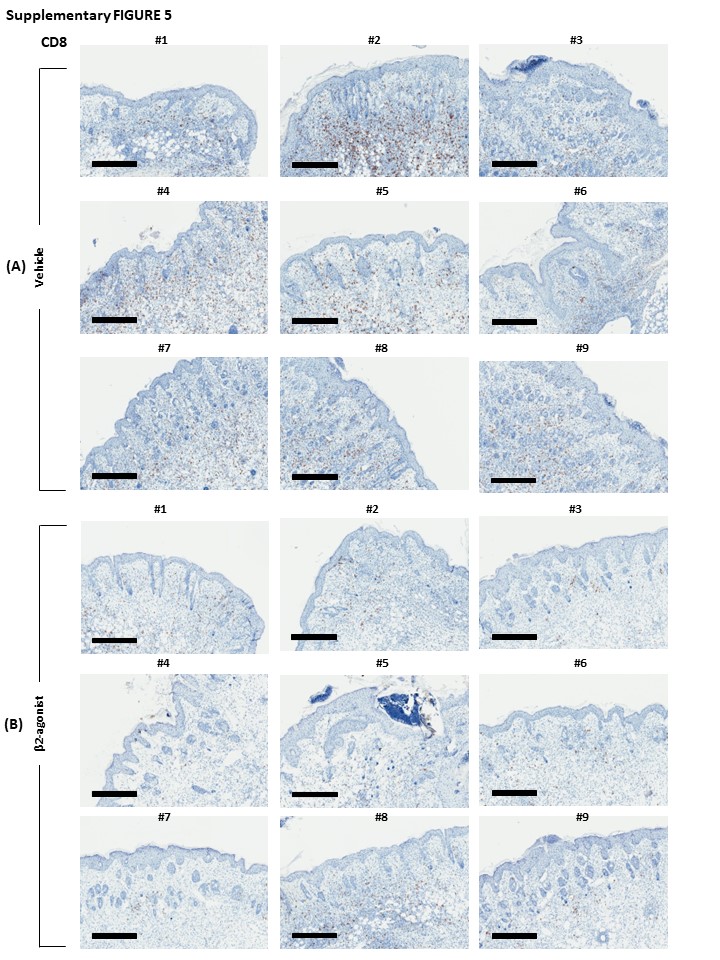

Supplement: Supplementary file 5 — FIGURE S5 Pathology findings of CD8 T‐cell infiltration 5 days after VCA. Representative IHC images of nine different transplanted grafts revealed CD8 T‐cell infiltration in the vehicle (A) and β2‐agonist (B) injected groups, scale bar: 400 µm [file CTM2-12-e996-s002.JPG]

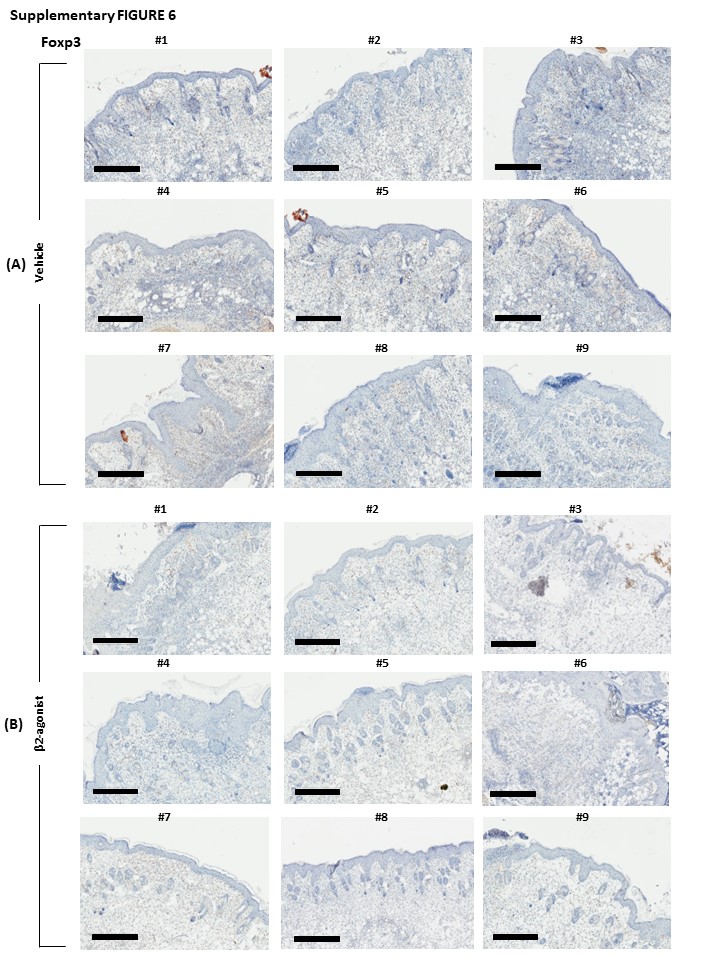

Supplement: Supplementary file 6 — FIGURE S6 Pathology findings of Foxp3 cell infiltration 5 days after VCA. Representative IHC images of nine different transplanted grafts revealed Foxp3 cell infiltration in the vehicle (A) and β2‐agonist (B) injected groups, scale bar: 400 µm [file CTM2-12-e996-s001.JPG]

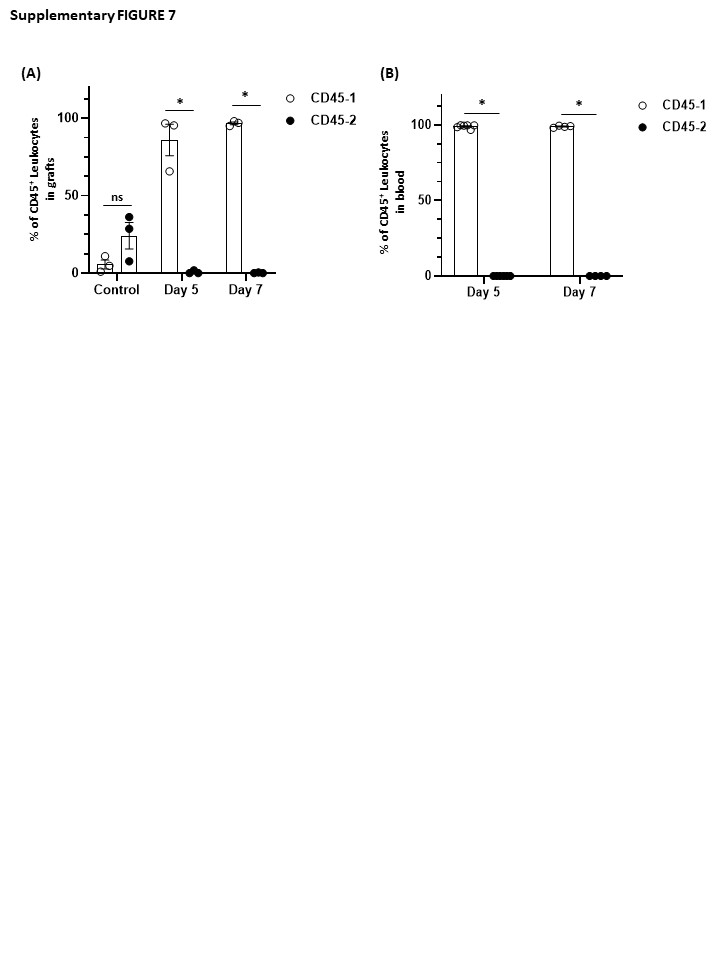

Supplement: Supplementary file 7 — FIGURE S7 The composition of donor's and recipient's leukocytes in donor grafts and recipient blood 5 and 7 days after VCA. (A and B) Source of infiltrated T cells in transplanted grafts and recipient's blood between recipient (CD45‐1) and donor (CD45‐2). Control, non‐vascularized grafts; n ≥ 3. ns, not significant; error bar, standard error of the mean. *p < .05 by Student's t test [file CTM2-12-e996-s010.JPG]

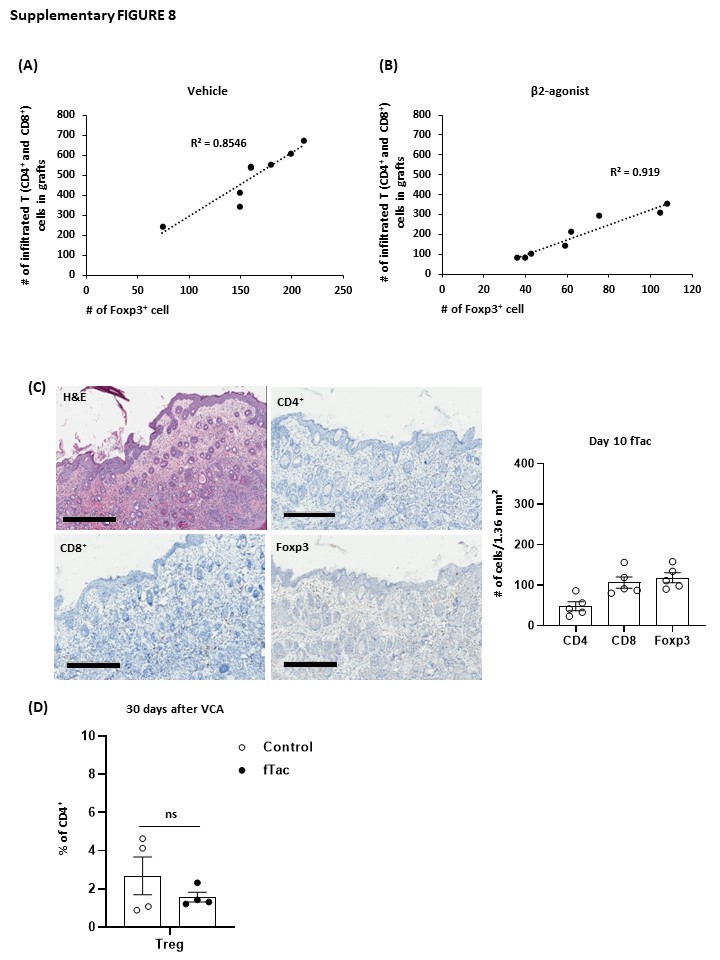

Supplement: Supplementary file 8 — FIGURE S8 The correlation of T‐cell infiltration and numbers of Foxp3+ cell in transplanted grafts after VCA. (A) Correlation analysis between numbers of infiltrated CD4/8 T and Foxp3+ cells in transplanted grafts (n = 8) 5 days after vehicle injections. (B) Correlation analysis between numbers of infiltrated CD4/8 T and Foxp3+ cells in transplanted grafts (n = 8) 5 days after β2‐agonist injections. The composition of Treg (CD4+CD25+Foxp3+) population was analysed with fTac injections after VCA. (C) Representative figure for H&E and IHC with numbers of CD4, CD8 and Foxp3 cells 10 days after VCA. Five fields from three grafts. Error bar, standard error of the mean; scale bar: 400 µm. (D) The Treg population was analysed with recipient's spleen 30 days after VCA. Control, a mouse without VCA; n = 4. ns, not significant; error bar, standard error of the mean [file CTM2-12-e996-s011.JPG]

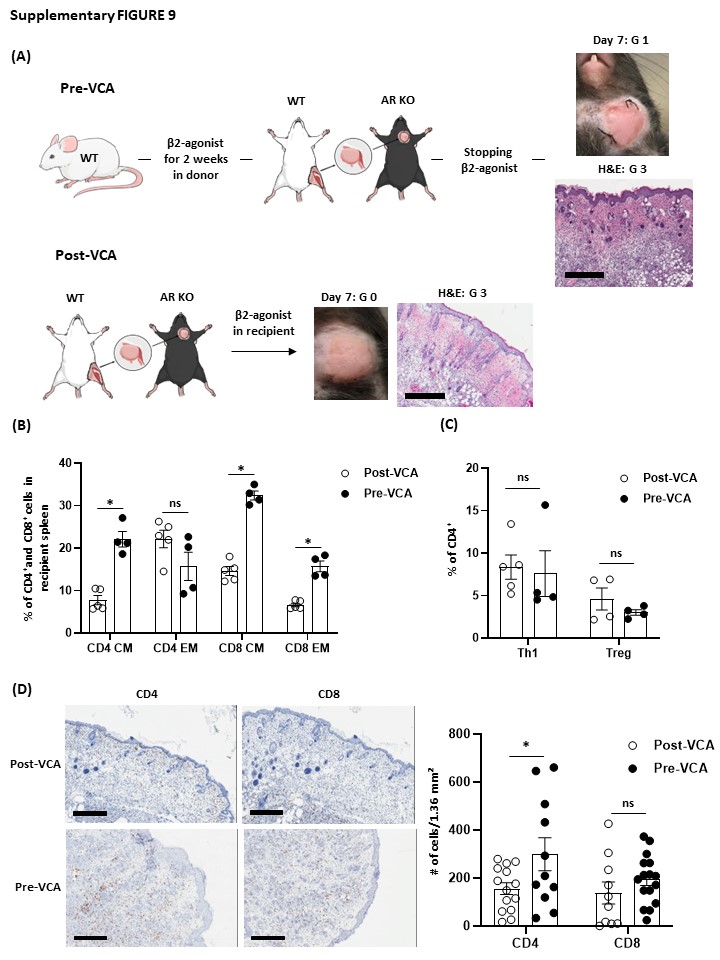

Supplement: Supplementary file 9 — FIGURE S9 Preconditioning in donors with β2‐AR agonist delays rejection responses through suppression of T‐cell trafficking in the grafts. (A) BALB/c donor mice were injected with β2‐agonist for 2 weeks before VCA (pre‐VCA), and then β2‐agonist treatment was stopped after the surgery in C57BL/6 AR KO recipients. Representative figures, scale bar: 400 µm. (B) Systemic compositions of CD4+/CD8+ CM and EM T‐cell populations 7 days after VCA. (C) Th1‐ and Treg‐cell populations in CD4+ T cells. n ≥ 4 mice. ns, not significant; error bar, standard error of the mean. *p < .05 by Student's t test. (D) Numbers of CD4 and CD8 positive cells in grafts 7 days after VCA. Over 10 fields from 3 mice/group. ns, not significant; error bar, standard error of the mean; scale bar: 400 µm. Data with empty circles (post‐VCA); historical data. *p < .05 by Student's t test [file CTM2-12-e996-s007.JPG]
