# Supplementary material for: Manipulating adrenergic stress receptor signalling to enhance immunosuppression and prolong survival of vascularized composite tissue transplants
Source: Clin Transl Med. 2022 Aug 22;12(8):e996. doi: 10.1002/ctm2.996 (PMC9394753; doi:10.1002/ctm2.996)
Supplement: Supplementary file 11 — Supporting Information [file CTM2-12-e996-s006.docx]

**Supplementary Materials:**

**Supplementary FIGURE 1** Gating Strategies for flow cytometry.

**Supplementary FIGURE 2** A schematic illustration of our vascularized composite tissue allotransplantation (VCA) model and the safety of a selective β_2_-adrenergic receptor (AR) agonist drug, terbutaline. (A) BALB/c and C57BL/6 strains were used as donors and recipients respectively. *En bloc* tissue composed of skin, subcutaneous fat, muscle, vessels and femur was transplanted to a recipient’s cervical area. (B) Systolic and diastolic blood pressure (BP) with heart rates (HR) were measured after everyday injection of with either vehicle (V; PBS) or β_2_-agonist (β; terbutaline; 2 mg/day). Mice were acclimated to the BP and HR measuring procedures for 10 days before recording data. Representative data between 2 different experiments, *n* = 5. (C) Concentrations of tacrolimus in plasma were analyzed 14 days after subcutaneous injections (24 hours after the last injection) with either a half dose of tacrolimus (hTac; 2 mg/kg/day) or a full dose of tacrolimus (fTac; 4 mg/kg/day). Representative data between 2 different experiments, *n* = 5, ns; not significant, **p* < 0.05 by Student’s t test, error bar; standard error of the mean.

**Supplementary FIGURE 3** Pathologic findings 5 and 7 days after VCA with either vehicle or β_2_-agonist injections. (A, B) Representative hematoxylin and eosin (H&E) images revealed epithelial dyskeratosis (arrowhead) and apoptosis (arrow) in the vehicle injected group (A; rejection grade 3) not in the β_2_-agonist injected group (B; rejection grade 2) 5 days after VCA, scale bar: 50 µm. (C, D) Representative figures for H&E and immunohistochemistry (IHC) with CD8, CD4, and Foxp3 antibodies either with vehicle or β_2_-agonist treatment for 7 days. (E) Numbers of CD4, CD8, and Foxp3 positive cells in grafts 7 days after VCA. 9 fields from 3 grafts per group, ns; not significant, error bar; standard error of the mean. (F) Compositions of CD4^+^/CD8^+^ central memory (CM) and effector memory (EM) T cell populations in transplanted grafts 7 days after VCA. Control; non-vascularized grafts, *n* = 5, ns; not significant, **p* < 0.05 by Student’s t test, error bar; standard error of the mean, scale bar; 400 µm.

**Supplementary FIGURE 4** Pathology findings of CD4 T cell infiltration 5 days after VCA. Representative IHC images of 9 different transplanted grafts revealed CD4 T cell infiltration in the vehicle (A) and β_2_-agonist (B) injected groups, scale bar; 400 μm.

**Supplementary FIGURE 5** Pathology findings of CD8 T cell infiltration 5 days after VCA. Representative IHC images of 9 different transplanted grafts revealed CD8 T cell infiltration in the vehicle (A) and β_2_-agonist (B) injected groups, scale bar; 400 μm.

**Supplementary FIGURE 6** Pathology findings of Foxp3 cell infiltration 5 days after VCA. Representative IHC images of 9 different transplanted grafts revealed Foxp3 cell infiltration in the vehicle (A) and β_2_-agonist (B) injected groups, scale bar; 400 μm.

**Supplementary FIGURE 7** The composition of donor’s and recipient’s leukocytes in donor grafts and recipient blood 5 and 7 days after VCA. (A, B) Source of infiltrated T cells in transplanted grafts and recipient’s blood between recipient (CD45-1) and donor (CD45-2). Control; non-vascularized grafts, *n* ≥ 3, ns; not significant, **p* < 0.05 by Student’s t test, error bar; standard error of the mean.

**Supplementary FIGURE 8** The correlation of T cell infiltration and numbers of Foxp3^+^ cell in transplanted grafts after VCA. (A) Correlation analysis between numbers of infiltrated CD4/8 T and Foxp3^+^ cells in transplanted grafts (*n* = 8) 5 days after vehicle injections. (B) Correlation analysis between numbers of infiltrated CD4/8 T and Foxp3^+^ cells in transplanted grafts (*n* = 8) 5 days after β_2_-agonist injections. The composition of Treg (CD4^+^CD25^+^Foxp3^+^) population was analyzed with fTac injections after VCA. (C) Representative figure for H&E and IHC with numbers of CD4, CD8 and Foxp3 cells 10 days after VCA. 5 fields from 3 grafts, error bar; standard error of the mean, scale bar; 400 μm. (D) The Treg population was analyzed with recipient’s spleen 30 days after VCA. Control; a mouse without VCA, *n* = 4, ns; not significant, error bar; standard error of the mean.

**Supplementary FIGURE 9** Preconditioning in donors with β_2_-AR agonist delays rejection responses through suppression of T cell trafficking in the grafts. (A) BALB/c donor mice were injected with β_2_-agonist for 2 weeks before VCA (Pre-VCA), and then β_2_-agonist treatment was stopped after the surgery in C57BL/6 AR KO recipients. Representative figures, scale bar; 400 μm. (B) Systemic compositions of CD4^+^/CD8^+^ CM and EM T cell populations 7 days after VCA. (C) Th1 and Treg cell populations in CD4^+^ T cells. *n* ≥ 4 mice **p* < 0.05 by Student’s t test, ns; not significant, error bar; standard error of the mean. (D) Numbers of CD4 and CD8 positive cells in grafts 7 days after VCA. Over 10 fields from 3 mice/group **p* < 0.05 by Student’s t test, ns; not significant, error bar; standard error of the mean, scale bar; 400 µm. Data with empty circles (Post-VCA); historical data.

**Supplementary TABLE 1** Two-way ANOVA analysis in the number of infiltrating CD4^+^ T and CD8^+^ T cells from day 5 to day 7.

**Supplementary TABLE 2** Two-way ANOVA analysis in changes of cytokine levels from day 5 to day 7.

**Supplementary TABLE 3** Two-way ANOVA analysis in changes of chemokine levels from day 5 to 7.
